# Supplementary material for: Mapping allosteric communications within individual proteins
Source: Nat Commun. 2020 Jul 31;11:3862. doi: 10.1038/s41467-020-17618-2 (PMC7395124; doi:10.1038/s41467-020-17618-2)
Supplement: Supplementary file 1 — Supplementary Information [file 41467_2020_17618_MOESM1_ESM.pdf]

SUPPLEMENTARY INFORMATION

Mapping Allosteric Communications within Proteins

Wang et al.

Supplementary Table 1. Information of the 20 proteins.

Protein name	PDB ID	Active site/Binding site ligand	Active Site Residues	Allosteric site ligand	Allosteric Site Residues	Length	Compositions
Caspase-1	2HBQ	PHQ	C/1,C/2,C/3,C/4,C/5,F/1,F/2,F/3,F/4,F/5	F1G	B/327,B/328,B/329,B/330,B/388,B/389,B/390,B/391,E/327,E/328,E/329,E/330,E/388,E/389,E/390,E/391	524	dimer
CheY	1F4V	FliM	D/1,D/2,D/3,D/4,D/5,D/6,D/7,D/8,D/9,D/10,D/11,D/12,D/13,D/14,D/15,D/16	BEF	A/57	147	monomer
Fructose 1,6-bisphosphatase	1EYI	F6P	A/339,B/339,C/339,D/339	AMP	A/27,A/28,A/29,A/30,A/31,B/27,B/28,B/29,B/30,B/31,C/27,C/28,C/29,C/30,C/31,D/27,D/28,D/29,D/30,D/31	1348	tetramer
Bacterial L-lactate dehydrogenase	1LTH	NAD	A/321,B/321,C/321,D/321	FBP	A/170,A/171,A/172,A/173,B/170,B/171,B/172,B/173,C/170,C/171,C/172,C/173,D/170,D/171,D/172,D/173	1260	tetramer
Protein-tyrosine phosphatase 1B	1PTY	PTR	A/323,A/324	BB3	A/189,A/192,A/193,A/196,A/287,A/288,A/291	302	monomer
Escherichia coli aspartate transcarbamoylase	1D09	CTP	B/999,D/999,F/999,H/999,J/999,L/999	PAL	A/52,A/54,A/55,A/105,A/167,A/134,E/54,E/52,E/55,E/105,E/167,E/134,I/52,I/54,I/55,I/105,I/167,I/134,G/59,G/62,G/292,G/293,G/296,G/136,K/59,K/62,K/292,K/293,K/296,K/136,K/137,C/59,C/62,C/292,C/293,C/296,C/136,C/137	2796	dodecamer
Thrombin	1SFQ	0G6	B/301,E/301	NAG	B/60,E/60	629	dimer
Monomeric Glucokinase (GCK)	1V4S	GLC	A/500	MRK	A/214,A/63,A/215,A/210,A/235,A/66,A/455	451	monomer
Sulfolobus solfataricus uracil phosphoribosyltransferase	1XTT	U5P	A/1250,D/4250,E/5250,H/8250	CTP	A/26,A/29,A/30,A/33,A/37,A/84,A/87,A/90,A/91,A/93,A/94,A/95,A/96,A/97,D/26,D/29,D/30,D/33,D/37,D/84,D/87,D/90,D/91,D/93,D/94,D/95,D/96,D/97,E/26,E/29,E/30,E/33,E/37,E/84,E/87,E/90,E/91,E/93,E/94,E/95,E/96,E/97,H/26,H/29,H/30,H/33,H/37,H/84,H/87,H/90,H/91,H/93,H/94,H/95,H/96,H/97	868	tetramer
H-Ras GTPase	3K8Y	GNP	A/528	ACT	A/97,A/100,A/101,A/106,A/107,A/108,A/109,A/111,A/137	172	monomer
Bovine glutamate dehydrogenase	6DHD	NAI	A/602,A/603,B/602,B/604,C/603,C/604,D/602,D/603,E/602,E/603,F/602,F/603	GTP	A/209,A/210,A/213,A/217,A/257,A/258,A/261,A/262,A/265,A/292,A/446,A/450,B/209,B/210,B/213,B/217,B/257,B/258,B/261,B/262,B/265,B/292,B/446,B/450,C/209,C/210,C/213,C/217,C/257,C/258,C/261,C/262,C/265,C/292,C/446,C/450,D/209,D/210,D/213,D/217,D/257,D/258,D/261,D/262,D/265,D/292,D/446,D/450,E/209,E/210,E/213,E/217,E/257,E/258,E/261,E/262,E/265,E/292,E/446,E/450,F/209,F/210,F/213,F/217,F/257,F/258,F/261,F/262,F/265,F/292,F/446,F/450	3030	hexamer
Lac repressor	1EFA	DNA	D/5,D/6,D/7,D/8,D/9,D/10,D/11,D/12,D/13,D/14,D/15,D/16,D/17,D/18,D/19,D/20,D/21,E/2,E/3,E/4,E/5,E/6,E/7,E/8,E/9,E/10,E/11,E/12,E/13,E/14,E/15,E/16,E/17,E/18	NPF	A/73,A/74,A/75,A/76,A/79,A/125,A/148,A/149,A/161,A/193,A/197,A/220,A/246,A/248,A/274,A/291,A/293,A/296,B/73,B/74,B/75,B/76,B/79,B/125,B/148,B/149,B/161,B/193,B/197,B/220,B/246,B/248,B/274,B/291,B/293,B/296	694	monomer

Phosphofructokinase	4PFK	F6P	A/323,B/323,C/323,D/323	PGA	A/154,A/185,A/187,A/211,A/213,A/214,A/215,B/154,B/185,B/187,B/211,B/213,B/214,B/215,C/154,C/185,C/187,C/211,C/213,C/214,C/215,D/154,D/185,D/187,D/211,D/213,D/214,D/215	1292	tetramer
PDK1 mutant bound to allosteric disulfide fragment activator 2A2	3ORZ	2A2	A/1	BI4	A/88,A/89,A/90,A/91,A/134,A/143,A/159,A/160,A/161,A/162,A/165,A/166,A/209,A/210,A/212,A/222,A/223	279	monomer
Chk1 Inhibitor	2BRG	AGY	A/900	DFY	A/84,A/85,A/86,A/87,A/88,A/89,A/90,A/15,A/16	259	monomer
Glycogen phosphorylase	7GPB	AMP	A/920,B/920,C/920,D/920	PLP	A/90,A/134,A/135,A/138,A/491,A/648,A/649,A/650,A/653,A/675,A/676,A/677,A/678,A/680,B/90,B/134,B/135,B/138,B/491,B/648,B/649,B/650,B/653,B/675,B/676,B/677,B/678,B/680,C/90,C/134,C/135,C/138,C/491,C/648,C/649,C/650,C/653,C/675,C/676,C/677,C/678,C/680,D/90,D/134,D/135,D/138,D/491,D/648,D/649,D/650,D/653,D/675,D/676,D/677,D/678,D/680	3311	tetramer
Glucosamine 6-phosphate deaminase	1HOT	PO4	A/267,B/268,C/267,D/268,E/267,F/268	16G	A/158,A/159,A/160,A/161,A/1,A/2,A/151,A/152,B/158,B/159,B/160,B/161,B/1,B/2,B/151,B/152,C/158,C/159,C/160,C/161,C/1,C/2,C/151,C/152,D/158,D/159,D/160,D/161,D/1,D/2,D/151,D/152	1608	hexamer
ATP sulfurylase from Penicillium chrysogenum	1I2D	ADX	A/574,B/576,C/578	PPS	A/405,A/517,A/526,A/527,A/528,A/529,A/530,A/534,A/434,A/437,A/451,A/454,A/455,B/405,B/517,B/526,B/527,B/528,B/529,B/530,B/534,B/434,B/437,B/451,B/454,B/455,C/405,C/517,C/526,C/527,C/528,C/529,C/530,C/534,C/434,C/437,C/451,C/454,C/455	1722	trimer
Potato tuber ADP-glucose pyrophosphorylase	1YP3	ATP	A/757,C/758	PMB	A/354,A/338,A/340,A/322,A/324,B/354,B/338,B/340,B/312,B/314,C/354,C/338,C/340,C/3228,D/14,D/354,D/338,D/340,D/322,D/324	1744	tetramer
Phosphoglycerate dehydrogenase	1YBA	AKG	A/413,B/412,C/412,D/412	SER	A/344,A/346,A/347,A/349,A/350,A/351,A/364,A/365,A/370,A/377,B/344,B/346,B/347,B/349,B/350,B/351,B/364,B/365,B/370,B/377,C/344,C/346,C/349,C/351,C/352,C/353,C/364,C/365,C/370,C/377,D/344,D/346,D/349,D/351,D/352,D/353,D/364,D/366,D/371,D/378	1648	tetramer

Supplementary Table 2. Comparison of the importance of critical residues of Caspase-1 between experiments and Ohm predictions.

Residue	Construct	k_{cat}/k_m ($M^{-1}S^{-1}$)	k_{cat}/k_m ratio	Residue Importance
-	Wild-type	5.3×10^4	1	-
**R286**	R286A	2.4×10^2	**230**	2.1729
S332	S332A	1.4×10^4	**3.7**	1.4419
S333	S333A	2.5×10^4	**2**	1.282
T334	T334A	3.1×10^4	**1.7**	0.2139
D336	D336A	3.8×10^4	**1.4**	0.254
N337	N337A	3.0×10^4	**1.8**	0.1468
S339	S339A	7.9×10^3	**6.7**	0.8329
T388	T388A	2.8×10^4	**1.9**	0.1467
**E390**	E390A	4.0×10^2	**130**	1.3006

Supplementary Table 3. URLs of all the results of the 20 proteins.

Protein name	PDB ID	URL[a]
Caspase-1	2HBQ	https://dokhlab.med.psu.edu/ohm/#/Task/641524720<br>https://dokhlab.med.psu.edu/ohm/#/Task/460875269<br>https://dokhlab.med.psu.edu/ohm/#/Task/560414097
CheY	1F4V	https://dokhlab.med.psu.edu/ohm/#/Task/350387717<br>https://dokhlab.med.psu.edu/ohm/#/Task/332337914<br>https://dokhlab.med.psu.edu/ohm/#/Task/528243153
Fructose 1,6-bisphosphatase	1EYI	https://dokhlab.med.psu.edu/ohm/#/Task/446114691<br>https://dokhlab.med.psu.edu/ohm/#/Task/563539950<br>https://dokhlab.med.psu.edu/ohm/#/Task/4155871
Bacterial L-lactate dehydrogenase	1LTH	https://dokhlab.med.psu.edu/ohm/#/Task/910885452<br>https://dokhlab.med.psu.edu/ohm/#/Task/584101415<br>https://dokhlab.med.psu.edu/ohm/#/Task/387361779
Protein-tyrosine phosphatase 1B	1PTY	https://dokhlab.med.psu.edu/ohm/#/Task/69122366<br>https://dokhlab.med.psu.edu/ohm/#/Task/237244894<br>https://dokhlab.med.psu.edu/ohm/#/Task/199003598
Escherichia coli aspartate transcarbamoylase	1D09	https://dokhlab.med.psu.edu/ohm/#/Task/499790283<br>https://dokhlab.med.psu.edu/ohm/#/Task/588293486<br>https://dokhlab.med.psu.edu/ohm/#/Task/354955558
Thrombin	1SFQ	https://dokhlab.med.psu.edu/ohm/#/Task/111087417<br>https://dokhlab.med.psu.edu/ohm/#/Task/513302697<br>https://dokhlab.med.psu.edu/ohm/#/Task/147026320
Monomeric Glucokinase (GCK)	1V4S	https://dokhlab.med.psu.edu/ohm/#/Task/358531358<br>https://dokhlab.med.psu.edu/ohm/#/Task/58021989<br>https://dokhlab.med.psu.edu/ohm/#/Task/664384935
Sulfolobus solfataricus uracil phosphoribosyltransferase	1XTT	https://dokhlab.med.psu.edu/ohm/#/Task/812307389<br>https://dokhlab.med.psu.edu/ohm/#/Task/430956726<br>https://dokhlab.med.psu.edu/ohm/#/Task/313593500
H-Ras GTPase	3K8Y	https://dokhlab.med.psu.edu/ohm/#/Task/228243128<br>https://dokhlab.med.psu.edu/ohm/#/Task/336028316<br>https://dokhlab.med.psu.edu/ohm/#/Task/169914149
Bovine glutamate dehydrogenase	6DHD	https://dokhlab.med.psu.edu/ohm/#/Task/308907842<br>https://dokhlab.med.psu.edu/ohm/#/Task/871281477<br>https://dokhlab.med.psu.edu/ohm/#/Task/881957688
Lac repressor	1EFA	https://dokhlab.med.psu.edu/ohm/#/Task/556930188

		https://dokhlab.med.psu.edu/ohm/#/Task/780117770<br>https://dokhlab.med.psu.edu/ohm/#/Task/326027222
Phosphofructokinase	4PFK	https://dokhlab.med.psu.edu/ohm/#/Task/572027245<br>https://dokhlab.med.psu.edu/ohm/#/Task/942954716<br>https://dokhlab.med.psu.edu/ohm/#/Task/375916349
PDK1 mutant bound to allosteric disulfide fragment activator 2A2	3ORZ	https://dokhlab.med.psu.edu/ohm/#/Task/455641212<br>https://dokhlab.med.psu.edu/ohm/#/Task/302646549<br>https://dokhlab.med.psu.edu/ohm/#/Task/454192001
Chk1 Inhibitor	2BRG	https://dokhlab.med.psu.edu/ohm/#/Task/306731174<br>https://dokhlab.med.psu.edu/ohm/#/Task/300339307<br>https://dokhlab.med.psu.edu/ohm/#/Task/966375409
Glycogen phosphorylase	7GPB	https://dokhlab.med.psu.edu/ohm/#/Task/18922679<br>https://dokhlab.med.psu.edu/ohm/#/Task/610662569<br>https://dokhlab.med.psu.edu/ohm/#/Task/719455894
Glucosamine 6-phosphate deaminase	1HOT	https://dokhlab.med.psu.edu/ohm/#/Task/182143714<br>https://dokhlab.med.psu.edu/ohm/#/Task/977570156<br>https://dokhlab.med.psu.edu/ohm/#/Task/928644384
ATP sulfurylase from Penicillium chrysogenum	1I2D	https://dokhlab.med.psu.edu/ohm/#/Task/898501793<br>https://dokhlab.med.psu.edu/ohm/#/Task/261452032<br>https://dokhlab.med.psu.edu/ohm/#/Task/732380935
Potato tuber ADP-glucose pyrophosphorylase	1YP3	https://dokhlab.med.psu.edu/ohm/#/Task/10381830<br>https://dokhlab.med.psu.edu/ohm/#/Task/118948547<br>https://dokhlab.med.psu.edu/ohm/#/Task/165632652
Phosphoglycerate dehydrogenase	1YBA	https://dokhlab.med.psu.edu/ohm/#/Task/186244623<br>https://dokhlab.med.psu.edu/ohm/#/Task/608980569<br>https://dokhlab.med.psu.edu/ohm/#/Task/462219307

[a] *The first, second, and third URL for each protein refers to the results of allosteric site prediction, allosteric pathway and critical residues identification, and inter-residue correlation calculation, respectively.*

Supplementary Table 4. Comparison of allosteric site prediction through Ohm and Amor's method.

Protein	Number of residue in allosteric sites	Ohm			Amor		
		Predicted Allosteric Hotspots	True Allosteric Hotspots	Allosteric Residue Hits	Predicted Allosteric Hotspots	True Allosteric Hotspots	Allosteric Residue Hits
2HBQ	16	1	1	14	8	4	14
1F4V	1	1	1	1	3	1	1
1EYI	20	14	6	20	41	0	0
1LTH	16	12	2	13	9	4	16
1PTY	7	1	1	3	10	1	4
1D09	38	13	7	37	36	10	20
1SFQ	2	3	1	1	7	0	0
1V4S	7	4	3	7	12	2	5
1XTT	56	3	3	45	9	2	2
3K8Y	9	1	1	9	3	1	1
6DHD	72	13	3	11	44	12	31
1EFA	36	6	4	33	11	4	14
4PFK	28	6	4	5	18	9	19
3ORZ	17	4	3	17	12	3	8
2BRG	9	3	1	2	8	2	6
7GPB	56	13	4	37	57	8	25
1HOT	32	9	4	32	27	3	5
1I2D	39	14	3	24	36	3	30
1YP3	20	8	2	4	37	3	10
1YBA	40	8	2	39	21	3	14

Supplementary Table 5. Comparison of residues ranked by importance of residue in Ohm pathways, ranked by betweenness centrality in Ohm pathways, and ranked by betweenness centrality in optimal pathways.

Protein	Residues ranked by importance of residue in Ohm pathways					Residues ranked by betweenness centrality in Ohm pathways					Residues ranked by betweenness centrality in optimal pathways				
2HBQ	D286	A286	D285	A285	A287	G1	H1	D286	A286	D285	A286	A285	D286	D285	C3
	C4	D287	F4	B390	E390	F4	A285	C4	F3	F5	E390	F3	B390	D258	D282
1F4V	A87	A106	D11	A89	A94	A130	D11	A87	A106	A89	A87	A108	D11	A59	A89
	A109	D15	D12	A95	D9	A95	D12	A94	D16	D15	A91	D3	A88	A90	A106
1EYI	D340	D342	B340	B342	C340	D342	B342	D339	B339	C339	C185	B243	A42	C44	C120
	A340	D118	B118	D121	C342	A339	C341	A341	C340	A340	C340	B120	D120	D243	A113
1LTH	B158	B83	B84	D158	A158	C320	D320	A158	A321	C321	A158	D158	C158	B158	A159
	C83	C84	C158	B39	A83	C158	B320	A320	C14	A14	A156	C54	A229	D155	D152
1PTY	A192	A191	A179	A215	A221	A300	A192	A191	A179	A323	A196	A195	A225	A221	A232
	A218	A224	A219	A217	A223	A215	A221	A218	A224	A219	A230	A253	A254		
1D09	F30	F17	F60	L90	L91	A1311	D999	L999	C1312	E1311	F77	F78	F81	F82	H77
	L60	F59	L11	L12	F58	J30	D59	L60	D18	J999	H78	H81	H82	I105	E105
1SFQ	B209	B208	E38	E39	B60	B209	B208	E38	E39	B700	B209	B208	E38	B60	B60
	B54	B53	B57	B195	E57	B301	B60	B57	B195	E301	B60	B59	B104	B53	E39
1V4S	A211	A205	A151	A207	A169	A501	A211	A500	A205	A151	A210	A206			
	A208	A168	A231	A206	A225	A207	A169	A208	A168	A231					
1XTT	H147	H148	A144	A105	H105	A1260	D4260	H8260	E5260	A1250	A29	A214	A210	D29	D214
	H119	H144	A148	A147	A119	H8250	E5250	E144	E105	A144	D210	E140	H140	A140	D140
3K8Y	A97	A96	A10	A16	A17	A719	A97	A96	A10	A528	A97	A96	A11		
	A168	A9	A35	A8	A34	A16	A17	A168	A9	A35					
6DHD	F86	B86	B85	F88	F122	D603	A603	A604	B603	F604	A209	B209	C209	D209	E209
	F123	F87	B488	B489	A392	F86	A209	D604	E604	B604	F209	C126	B208	B203	D208
1EFA	B125	B124	B111	A125	A124	B902	A901	E14	E13	B125	A149	A124	A111	A112	B48
	B48	B112	A111	E14	A59	B124	B111	A125	A124	A59	B79	B83	B87	B92	B62
4PFK	C249	D154	C252	B154	A249	C323	A323	C325	D325	D154	A154	C126	B154	C154	D154
	D156	D155	C154	A154	B155	C154	B323	B325	A325	A154	A126	D252	B126	D126	C153
3ORZ	A159	A145	A148	A131	A158	A360	A159	A1	A145	A148	A159	A145	A148		
	A115	A149	A155	A118	A127	A131	A158	A115	A149	A155					
2BRG	A137	A96	A94	A93	A97	A1270	A900	A137	A96	A94	A91	A93			
	A138	A91	A92	A98	A133	A93	A97	A138	A91	A92					
7GPB	B44	B45	B42	B649	B651	C920	A920	C310	C309	B999	A650	D309	B90	B242	C310
	B93	B126	B125	B48	B47	C71	C999	C314	A999	A71	C90	A91	A241	A242	A651
1HOT	B158	B160	B161	F258	F259	A219	A228	A227	A221	C219	E152	A153	E251	D152	A251
	F4	F238	F12	F11	F10	A194	E251	B227	A218	A217	A158	A155	B158	A232	D251
1I2D	B197	B198	B333	B332	B292	B576	A575	A477	A478	A400	C565	B207	C478	C400	C399
	B291	B290	B295	B209	B294	C579	C477	C478	C400	B577	A478	A400	A399	C366	C367
1YP3	A26	A120	A25	A27	A43	A757	C758	A26	C27	A120	B314	B83	C1005	C134	C131
	A28	A29	A1020	A144	A338	A25	C26	C28	A43	A27	C73	B338	B337	A144	A340
1YBA	A119	A115	A294	A127	A84	A84	B412	A119	A115	A294	D295	A112	A108	C311	A344
	B60	B61	A102	A103	A142	A127	A103	A102	A141	B295	A343	A315	A311	A351	D368

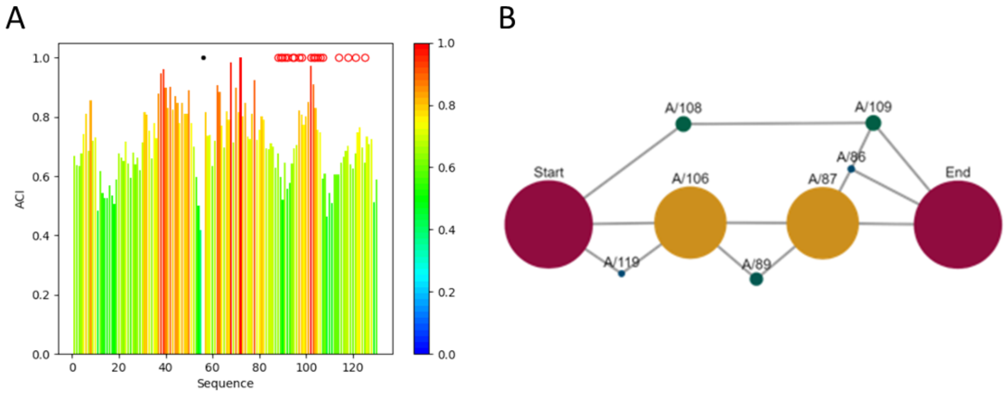

Supplementary Figure 1. Allosteric analysis of CheY when the active site and allosteric site are switched. (A) ACI of residues in CheY (1FQW). The black dot refers to D57 and the red circles refer to residues 89, 90, 91, 92, 93, 95, 96, 98, 99, 103, 104, 105, 106, 107, 108, 115 ,119, 122, and 126. (B) The allosteric pathways from the FliM helix to D57.

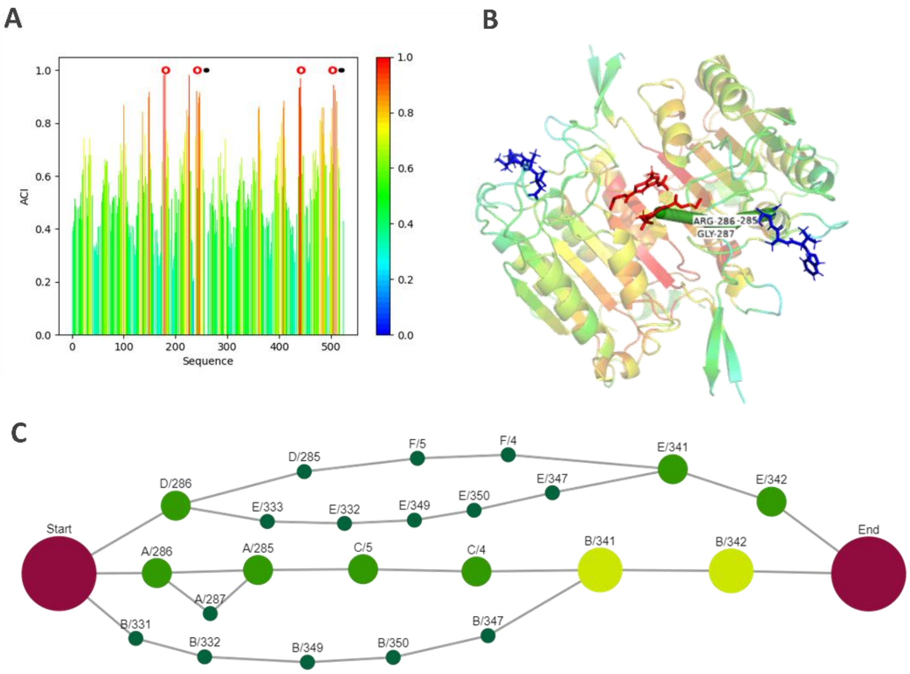

Supplementary Figure 2. Caspase-1 (PDB ID: 2HBQ). (A) ACI calculated by Ohm. (B) Tertiary structure rendered by ACI. The ligand in the active site is colored by blue and the ligand in the allosteric site is colored by red. An identified pathway is shown as green cylinders. (C) Critical residues involved in core allosteric pathways.

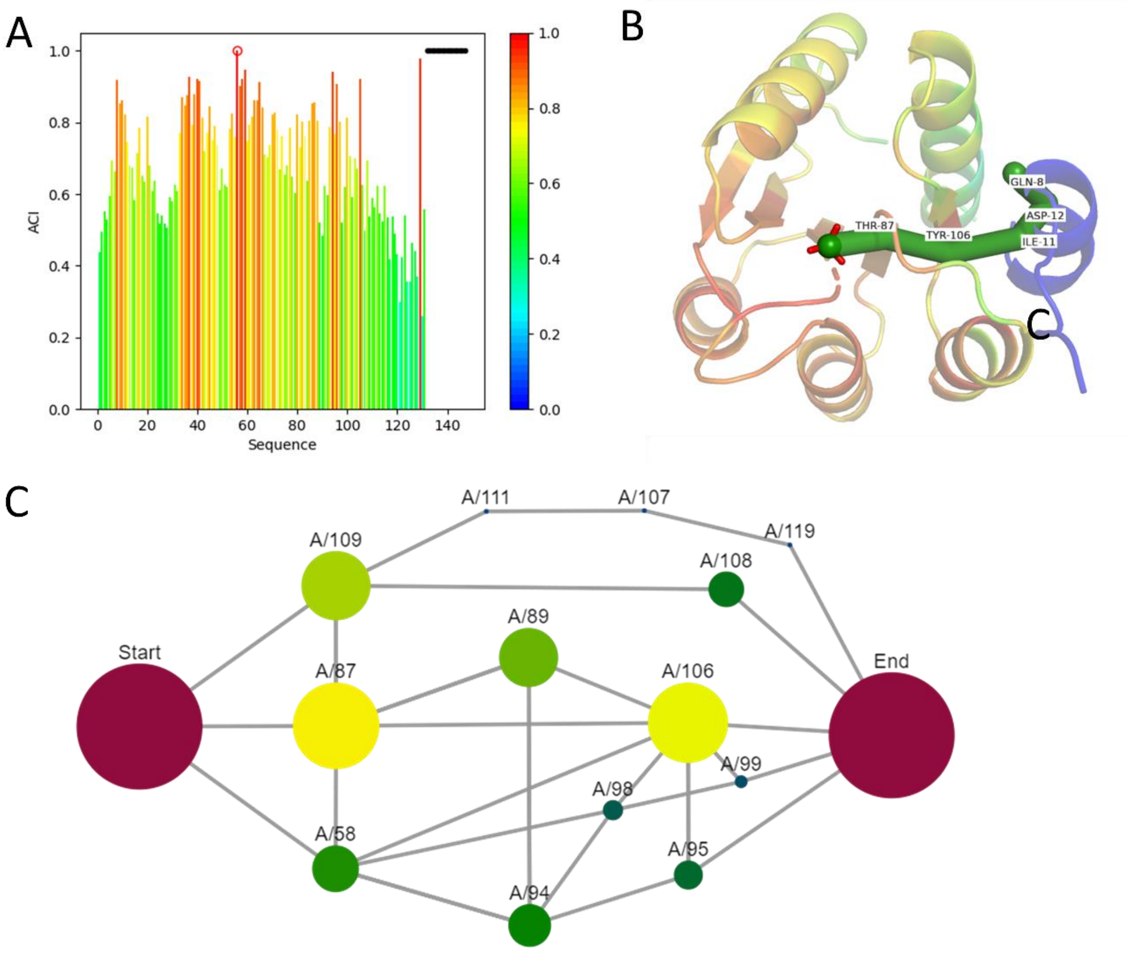

Supplementary Figure 3. CheY (PDB ID: 1F4V). (A) ACI calculated by Ohm. (B) Tertiary structure rendered by ACI. The ligand in the active site is colored by blue and the ligand in the allosteric site is colored by red. An identified pathway is shown as green cylinders. (C) Critical residues involved in core allosteric pathways.

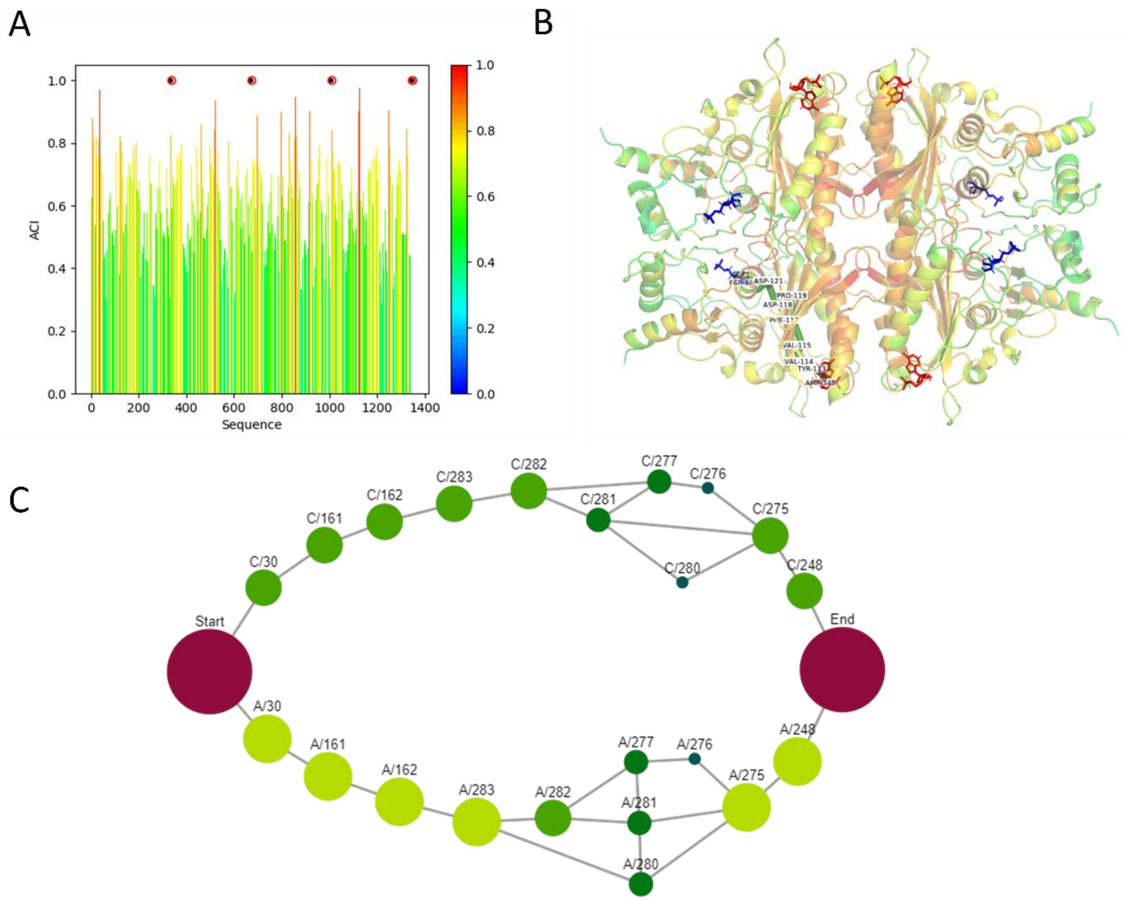

Supplementary Figure 4. Fructose 1,6-bisphosphatase (PDB ID: 1EYI). (A) ACI calculated by Ohm. (B) Tertiary structure rendered by ACI. The ligand in the active site is colored by blue and the ligand in the allosteric site is colored by red. An identified pathway is shown as green cylinders. (C) Critical residues involved in core allosteric pathways.

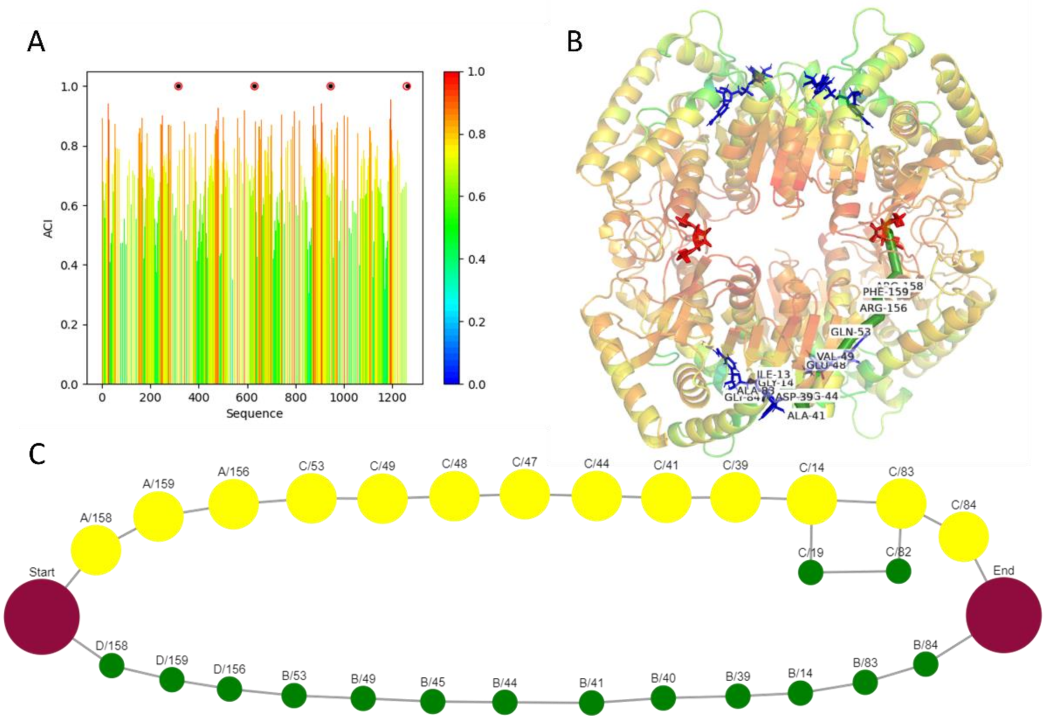

Supplementary Figure 5. Bacterial L-lactate dehydrogenase (PDB ID: 1LTH). (A) ACI calculated by Ohm. (B) Tertiary structure rendered by ACI. The ligand in the active site is colored by blue and the ligand in the allosteric site is colored by red. An identified pathway is shown as green cylinders. (C) Critical residues involved in core allosteric pathways.

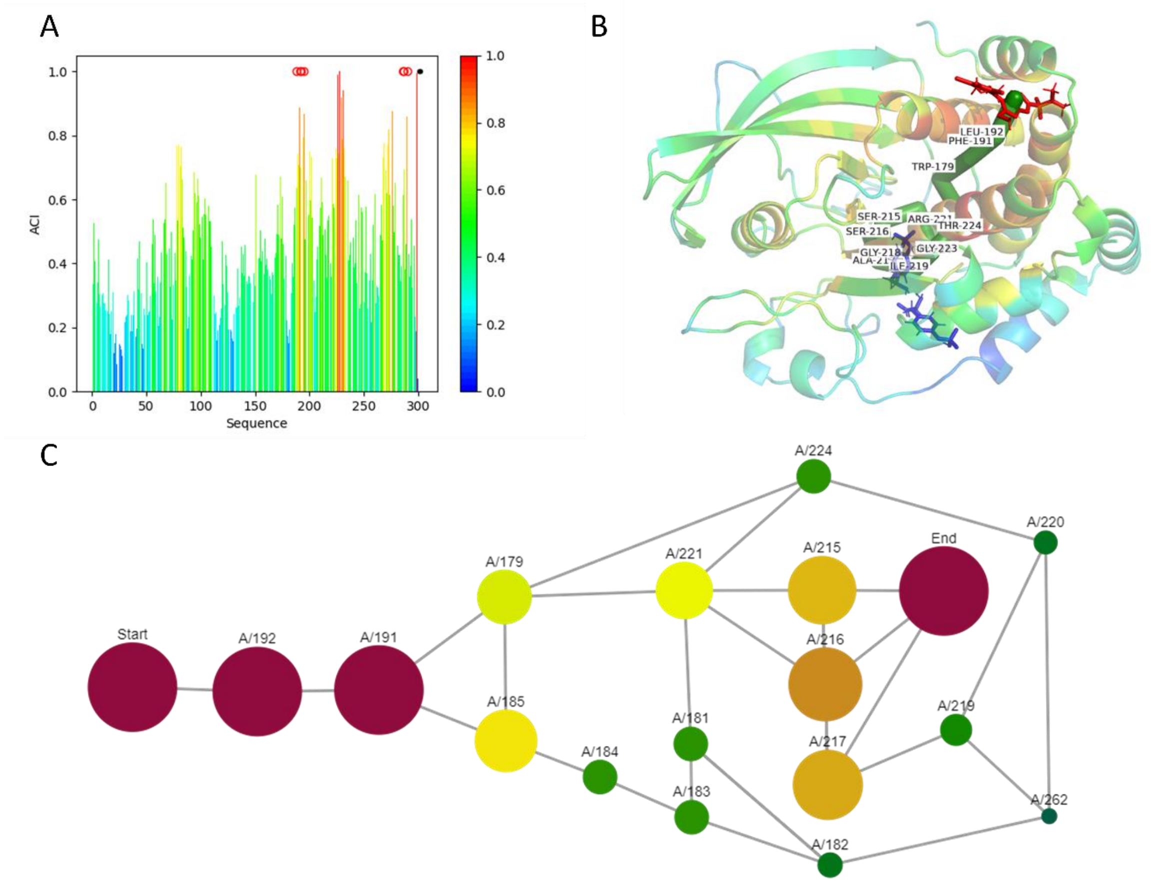

Supplementary Figure 6. Protein-tyrosine phosphatase 1B (PDB ID: 1PTY). (A) ACI calculated by Ohm. (B) Tertiary structure rendered by ACI. The ligand in the active site is colored by blue and the ligand in the allosteric site is colored by red. An identified pathway is shown as green cylinders. (C) Critical residues involved in core allosteric pathways.

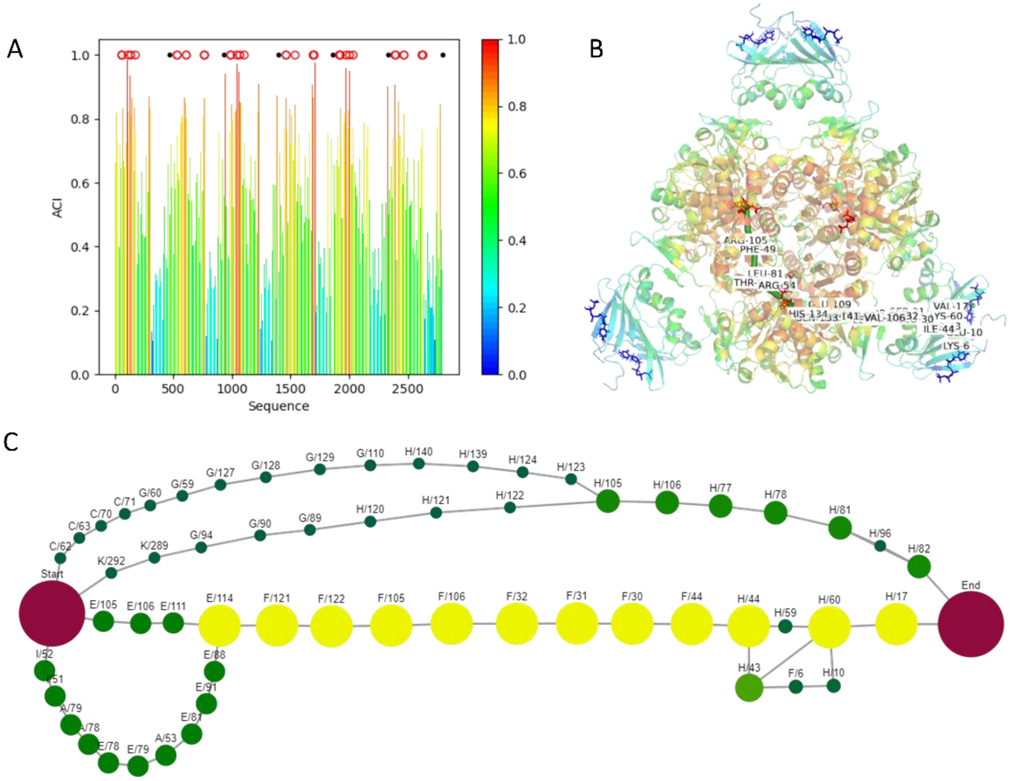

Supplementary Figure 7. Escherichia coli aspartate transcarbamoylase (PDB ID: 1D09). (A) ACI calculated by Ohm. (B) Tertiary structure rendered by ACI. The ligand in the active site is colored by blue and the ligand in the allosteric site is colored by red. An identified pathway is shown as green cylinders. (C) Critical residues involved in core allosteric pathways.

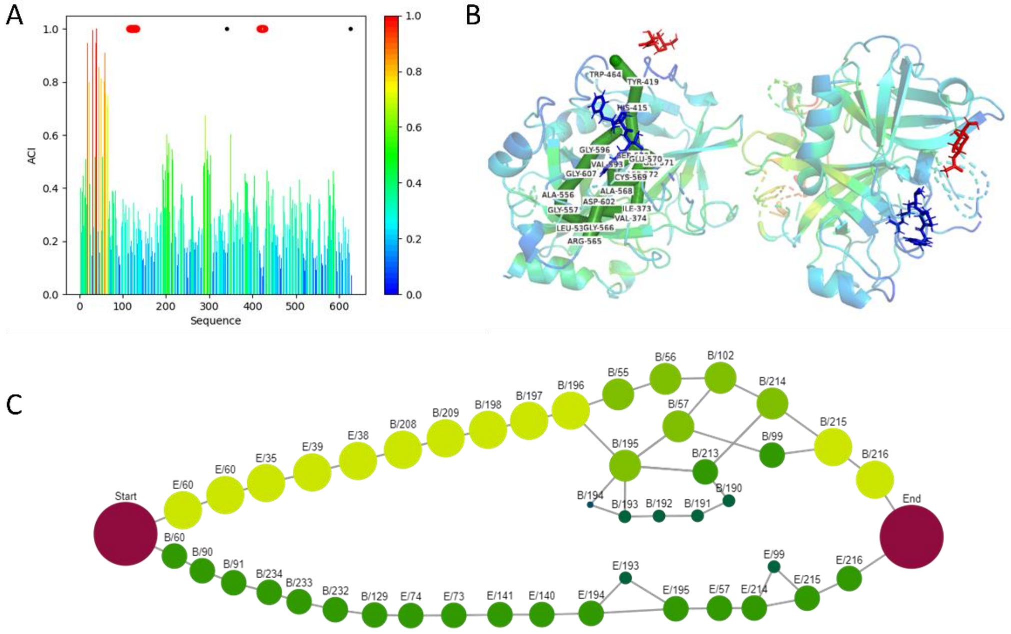

Supplementary Figure 8. Thrombin (PDB ID: 1SFQ). (A) ACI calculated by Ohm. (B) Tertiary structure rendered by ACI. The ligand in the active site is colored by blue and the ligand in the allosteric site is colored by red. An identified pathway is shown as green cylinders. (C) Critical residues involved in core allosteric pathways.

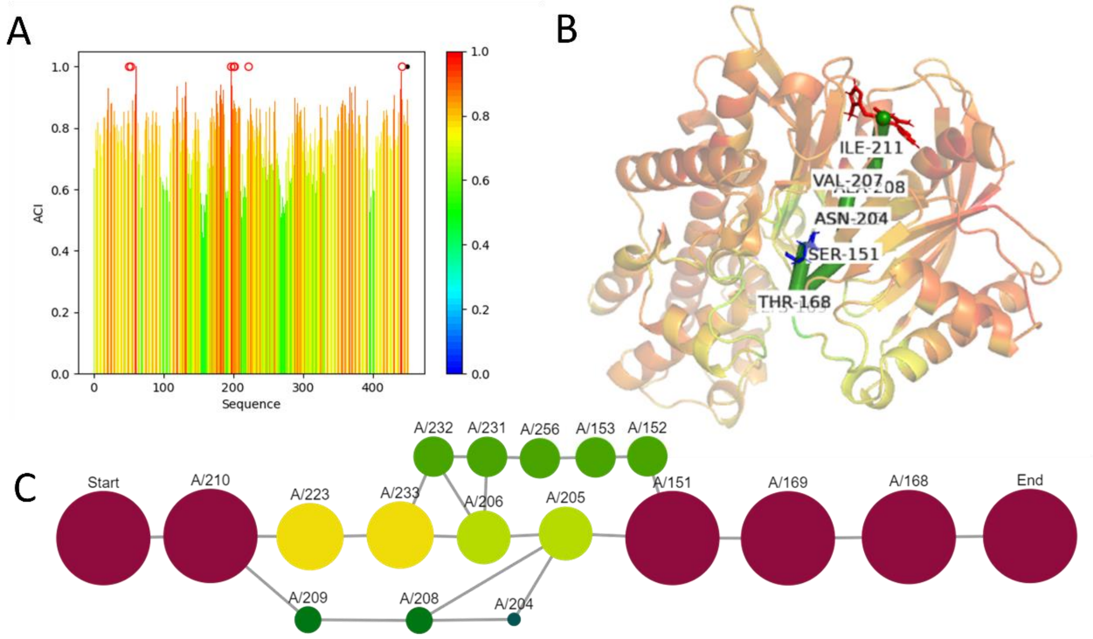

Supplementary Figure 9. Monomeric Glucokinase (GCK) (PDB ID: 1V4S). (A) ACI calculated by Ohm. (B) Tertiary structure rendered by ACI. The ligand in the active site is colored by blue and the ligand in the allosteric site is colored by red. An identified pathway is shown as green cylinders. (C) Critical residues involved in core allosteric pathways.

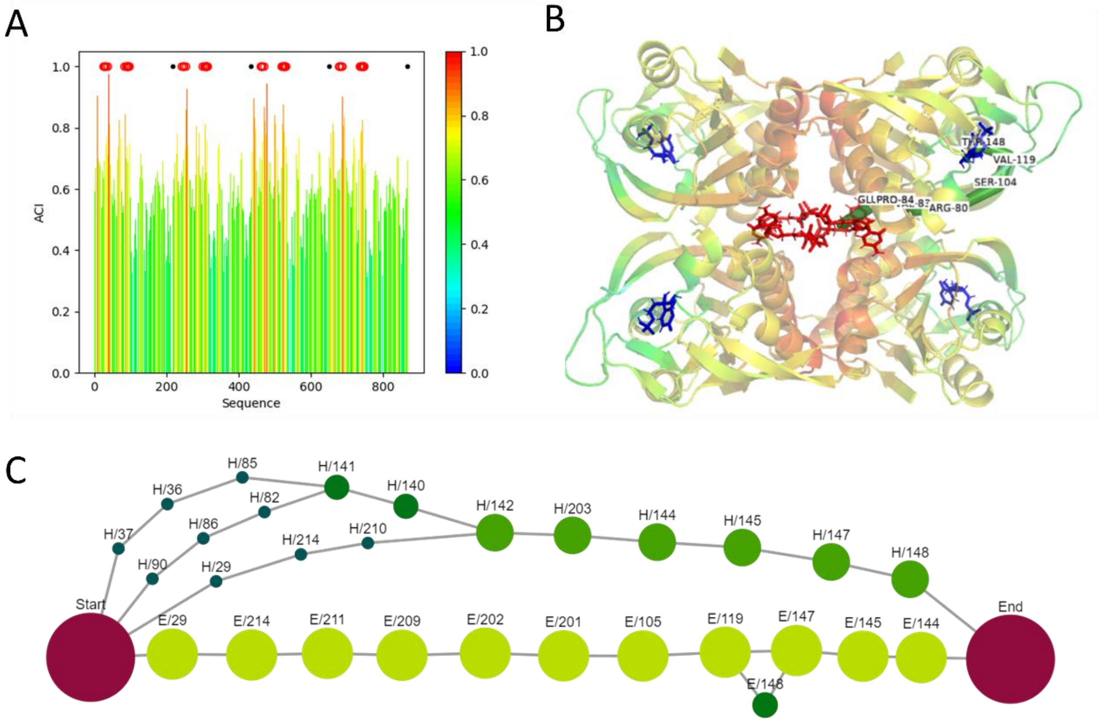

Supplementary Figure 10. Sulfolobus solfataricus uracil phosphoribosyltransferase (PDB ID: 1XTT). (A) ACI calculated by Ohm. (B) Tertiary structure rendered by ACI. The ligand in the active site is colored by blue and the ligand in the allosteric site is colored by red. An identified pathway is shown as green cylinders. (C) Critical residues involved in core allosteric pathways.

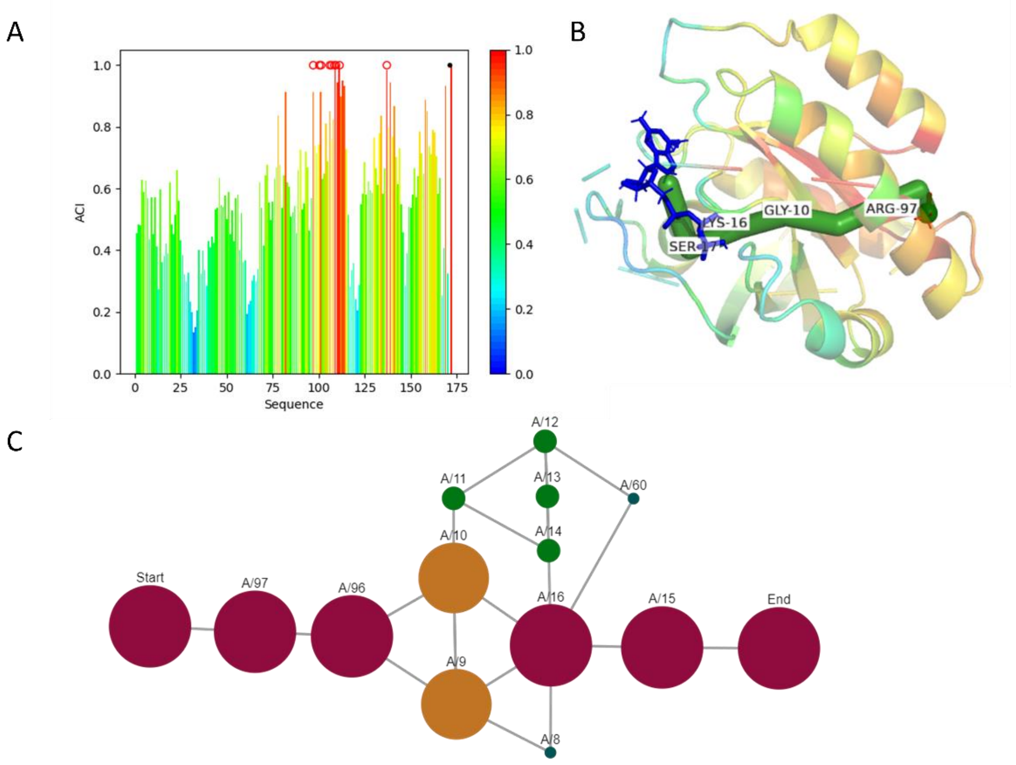

Supplementary Figure 11. H-Ras GTPase (PDB ID: 3K8Y). (A) ACI calculated by Ohm. (B) Tertiary structure rendered by ACI. The ligand in the active site is colored by blue and the ligand in the allosteric site is colored by red. An identified pathway is shown as green cylinders. (C) Critical residues involved in core allosteric pathways.

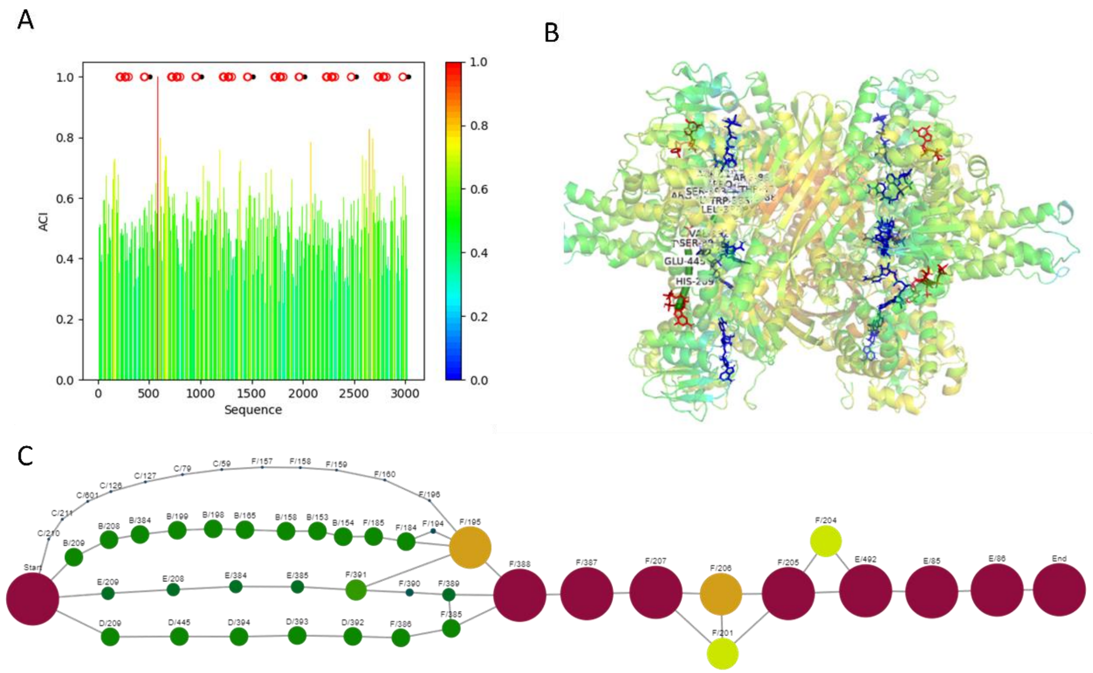

Supplementary Figure 12. Bovine glutamate dehydrogenase (PDB ID: 6DHD). (A) ACI calculated by Ohm. (B) Tertiary structure rendered by ACI. The ligand in the active site is colored by blue and the ligand in the allosteric site is colored by red. An identified pathway is shown as green cylinders. (C) Critical residues involved in core allosteric pathways.

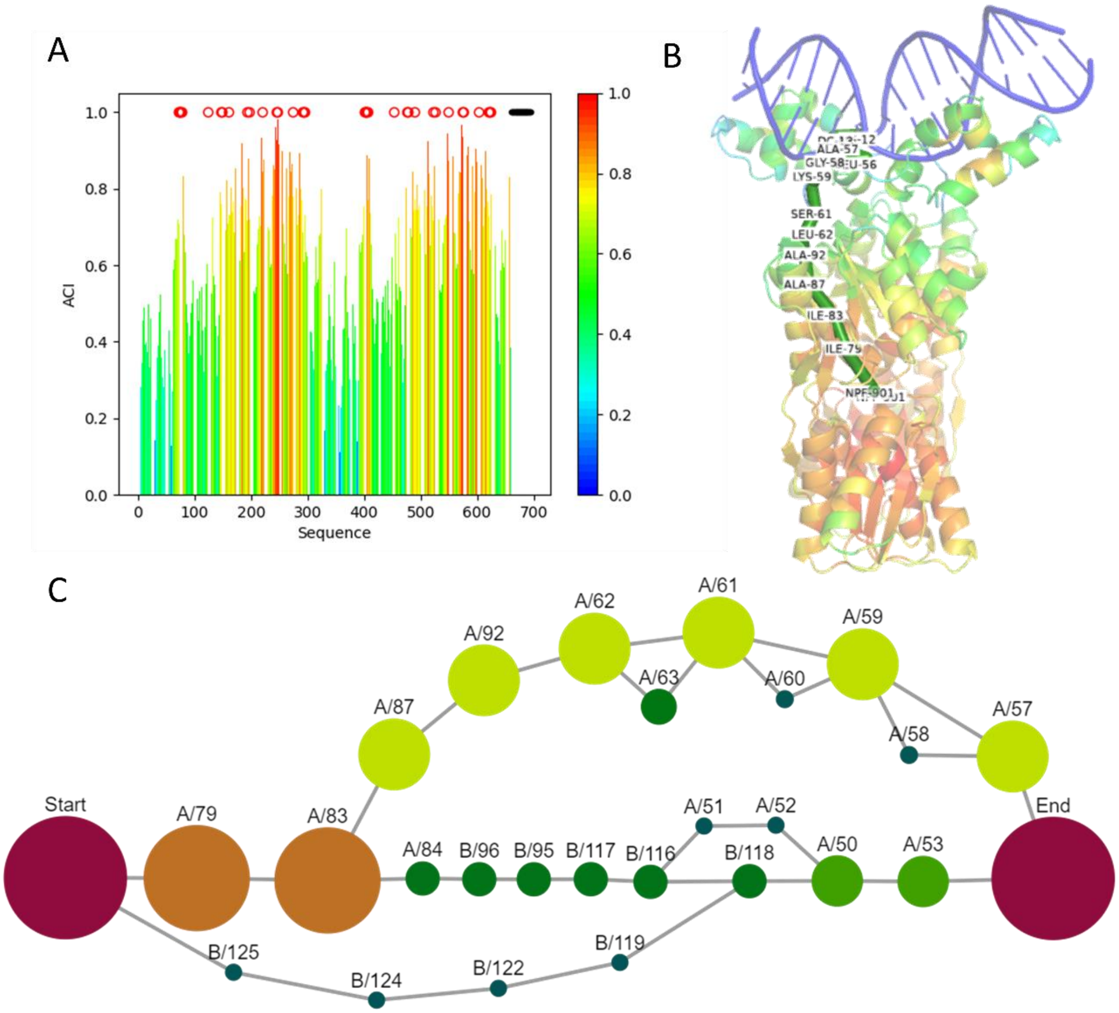

Supplementary Figure 13. Lac repressor (PDB ID: 1EFA). (A) ACI calculated by Ohm. (B) Tertiary structure rendered by ACI. The ligand in the active site is colored by blue and the ligand in the allosteric site is colored by red. An identified pathway is shown as green cylinders. (C) Critical residues involved in core allosteric pathways.

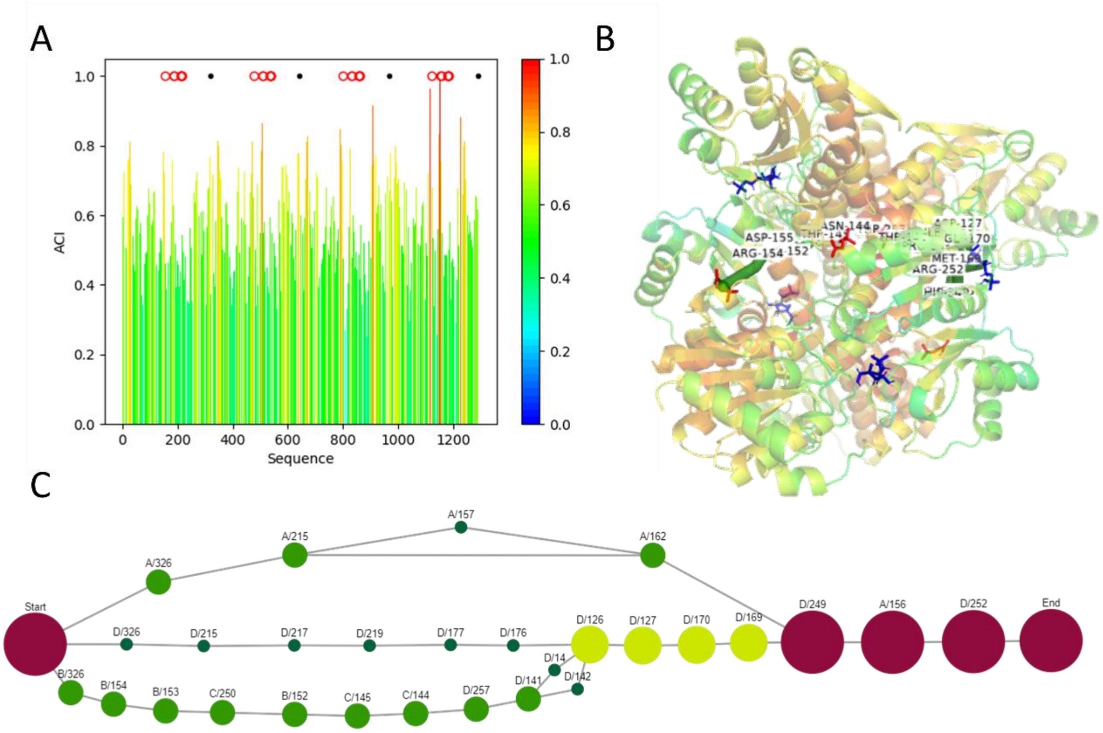

Supplementary Figure 14. Phosphofructokinase (PDB ID: 4PFK). (A) ACI calculated by Ohm. (B) Tertiary structure rendered by ACI. The ligand in the active site is colored by blue and the ligand in the allosteric site is colored by red. An identified pathway is shown as green cylinders. (C) Critical residues involved in core allosteric pathways.

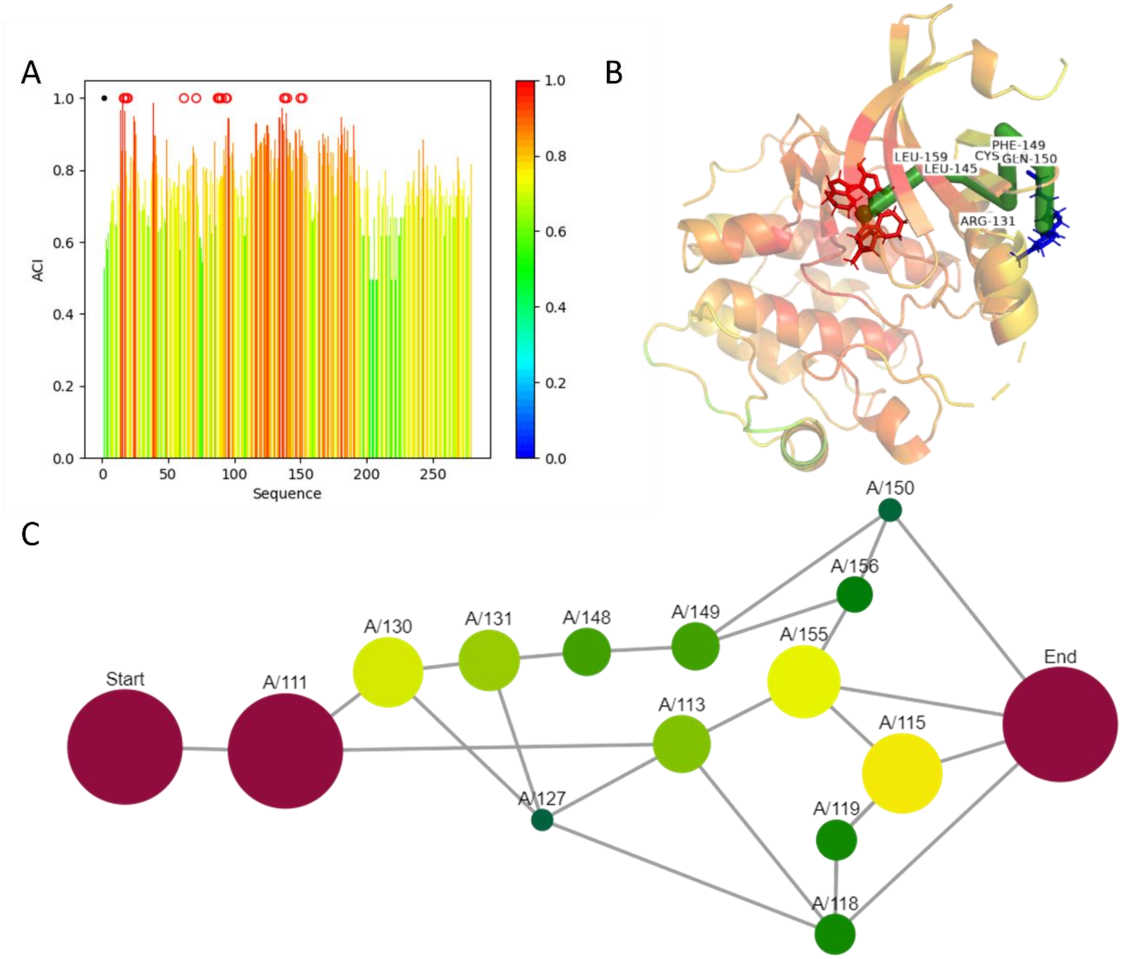

Supplementary Figure 15. PDK1 mutant bound to allosteric disulfide fragment activator 2A2 (PDB ID: 3ORZ). (A) ACI calculated by Ohm. (B) Tertiary structure rendered by ACI. The ligand in the active site is colored by blue and the ligand in the allosteric site is colored by red. An identified pathway is shown as green cylinders. (C) Critical residues involved in core allosteric pathways.

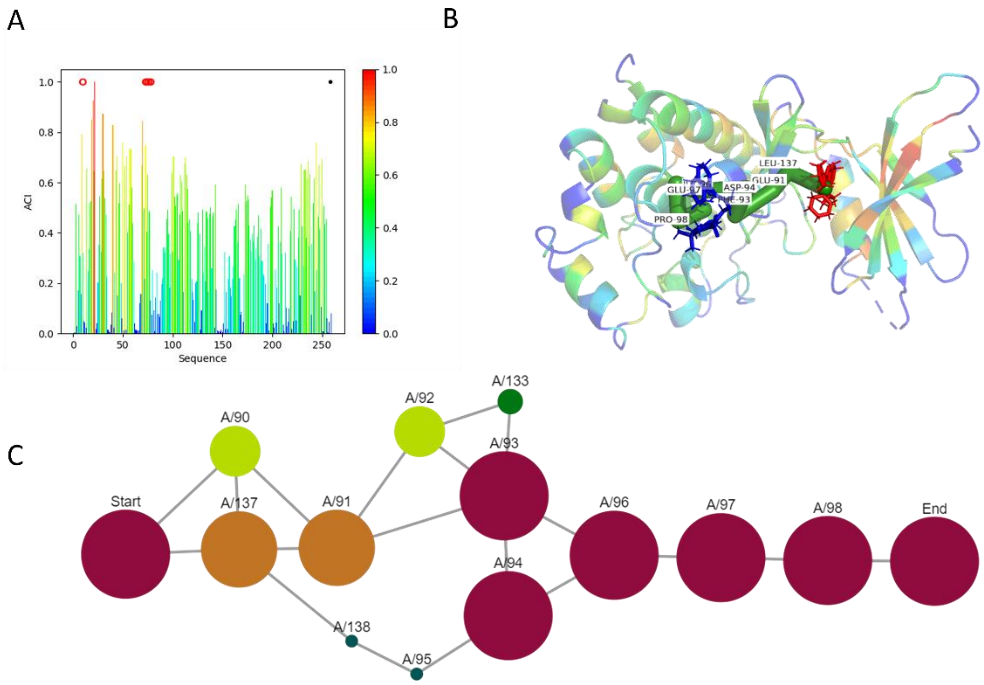

Supplementary Figure 16. Chk1 Inhibitor (PDB ID: 2BRG). (A) ACI calculated by Ohm. (B) Tertiary structure rendered by ACI. The ligand in the active site is colored by blue and the ligand in the allosteric site is colored by red. An identified pathway is shown as green cylinders. (C) Critical residues involved in core allosteric pathways.

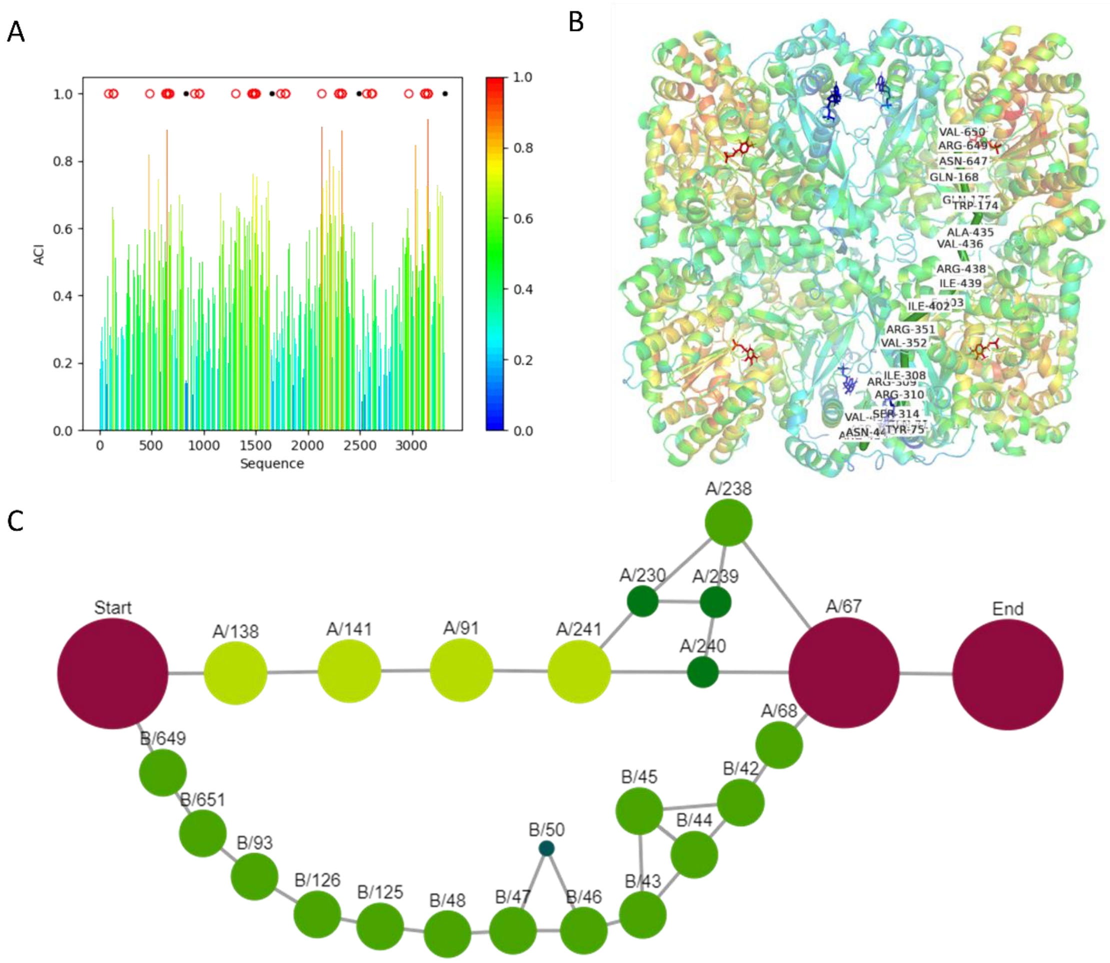

Supplementary Figure 17. Glycogen phosphorylase (PDB ID: 7GPB). (A) ACI calculated by Ohm. (B) Tertiary structure rendered by ACI. The ligand in the active site is colored by blue and the ligand in the allosteric site is colored by red. An identified pathway is shown as green cylinders. (C) Critical residues involved in core allosteric pathways.

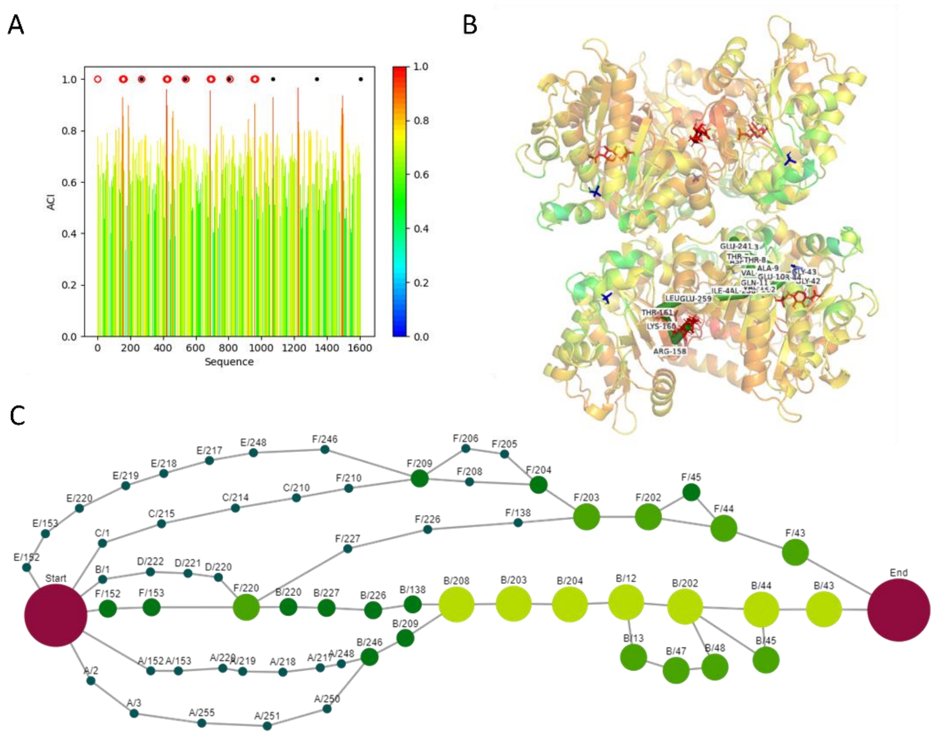

Supplementary Figure 18. Glucosamine 6-phosphate deaminase (PDB ID: 1HOT). (A) ACI calculated by Ohm. (B) Tertiary structure rendered by ACI. The ligand in the active site is colored by blue and the ligand in the allosteric site is colored by red. An identified pathway is shown as green cylinders. (C) Critical residues involved in core allosteric pathways.

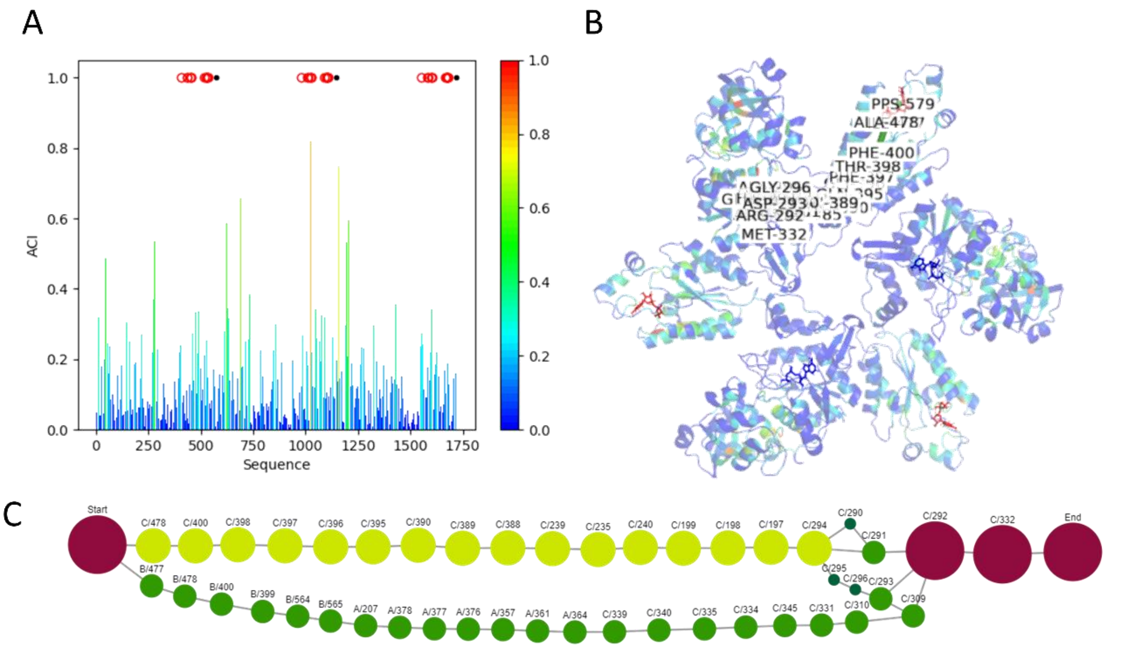

Supplementary Figure 19. ATP sulfurylase from Penicillium chrysogenum (PDB ID: 1I2D). (A) ACI calculated by Ohm. (B) Tertiary structure rendered by ACI. The ligand in the active site is colored by blue and the ligand in the allosteric site is colored by red. An identified pathway is shown as green cylinders. (C) Critical residues involved in core allosteric pathways.

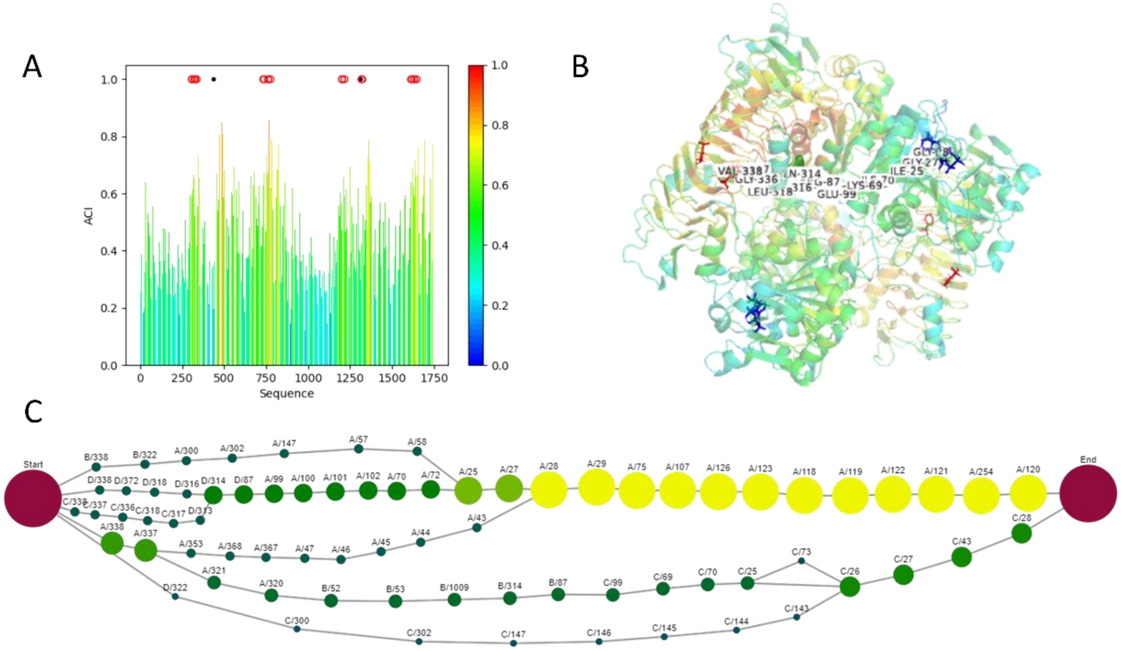

Supplementary Figure 20. Potato tuber ADP-glucose pyrophosphorylase (PDB ID: 1YP3). (A) ACI calculated by Ohm. (B) Tertiary structure rendered by ACI. The ligand in the active site is colored by blue and the ligand in the allosteric site is colored by red. An identified pathway is shown as green cylinders. (C) Critical residues involved in core allosteric pathways.

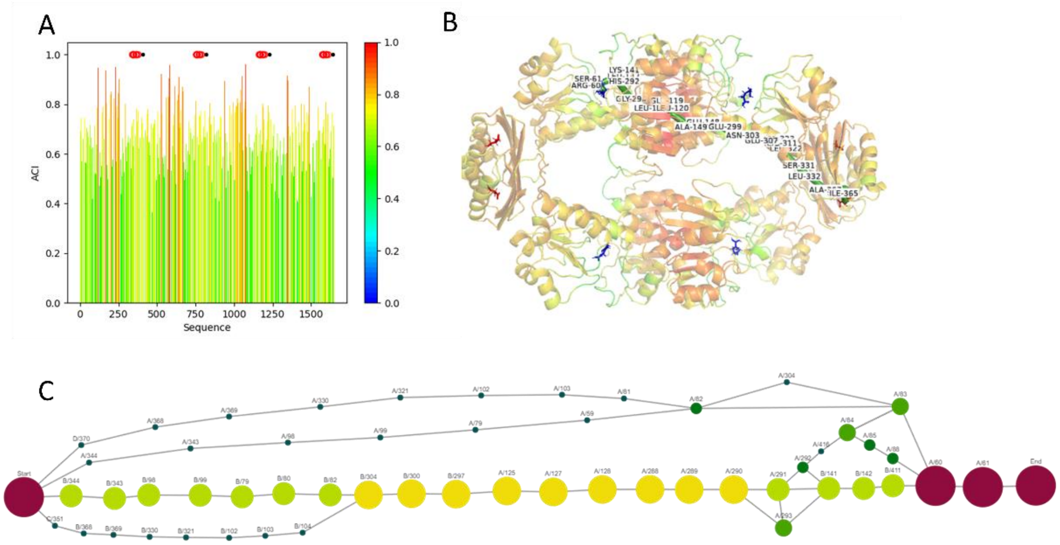

Supplementary Figure 21. Phosphoglycerate dehydrogenase (PDB ID: 1YBA). (A) ACI calculated by Ohm. (B) Tertiary structure rendered by ACI. The ligand in the active site is colored by blue and the ligand in the allosteric site is colored by red. An identified pathway is shown as green cylinders. (C) Critical residues involved in core allosteric pathways.

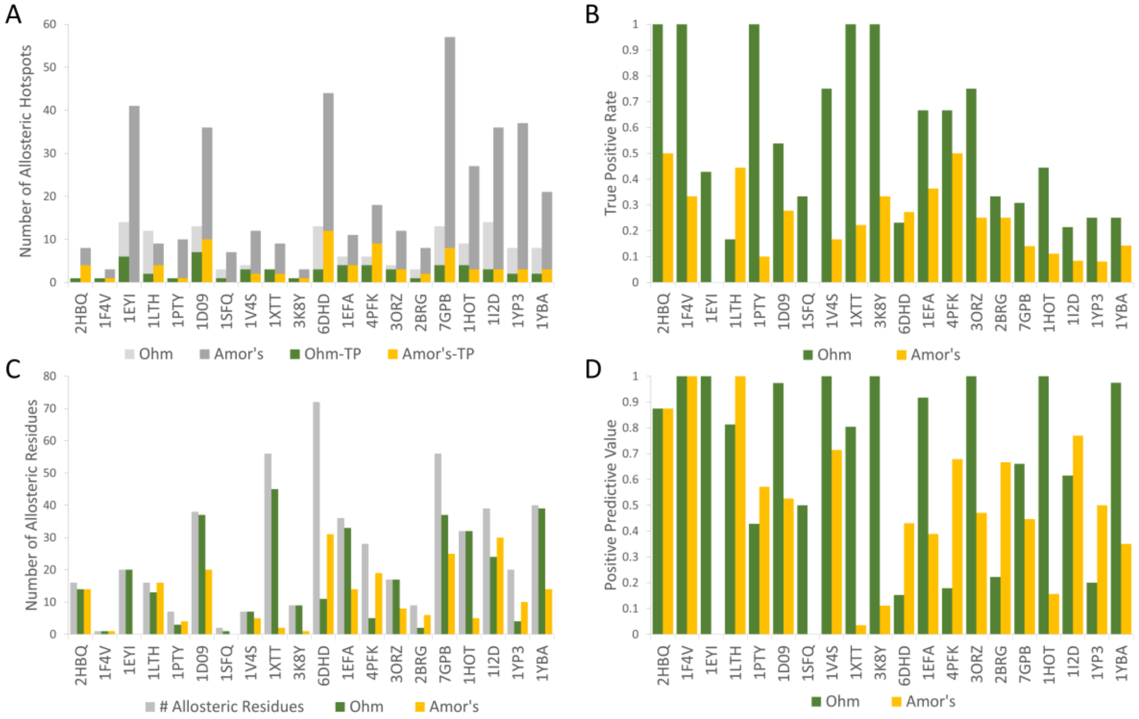

Supplementary Figure 22. Comparison of Ohm to Amor's method in allosteric site prediction on the 20 proteins dataset. (A) The number of true positive and all hotspots predicted by Ohm and Amor's method. (B) TPR of hotspots identified by Ohm and Amor's method. (C) The number of allosteric residues identified by Ohm and Amor's method. (D) PPV of allosteric residues identified by Ohm and Amor's method.

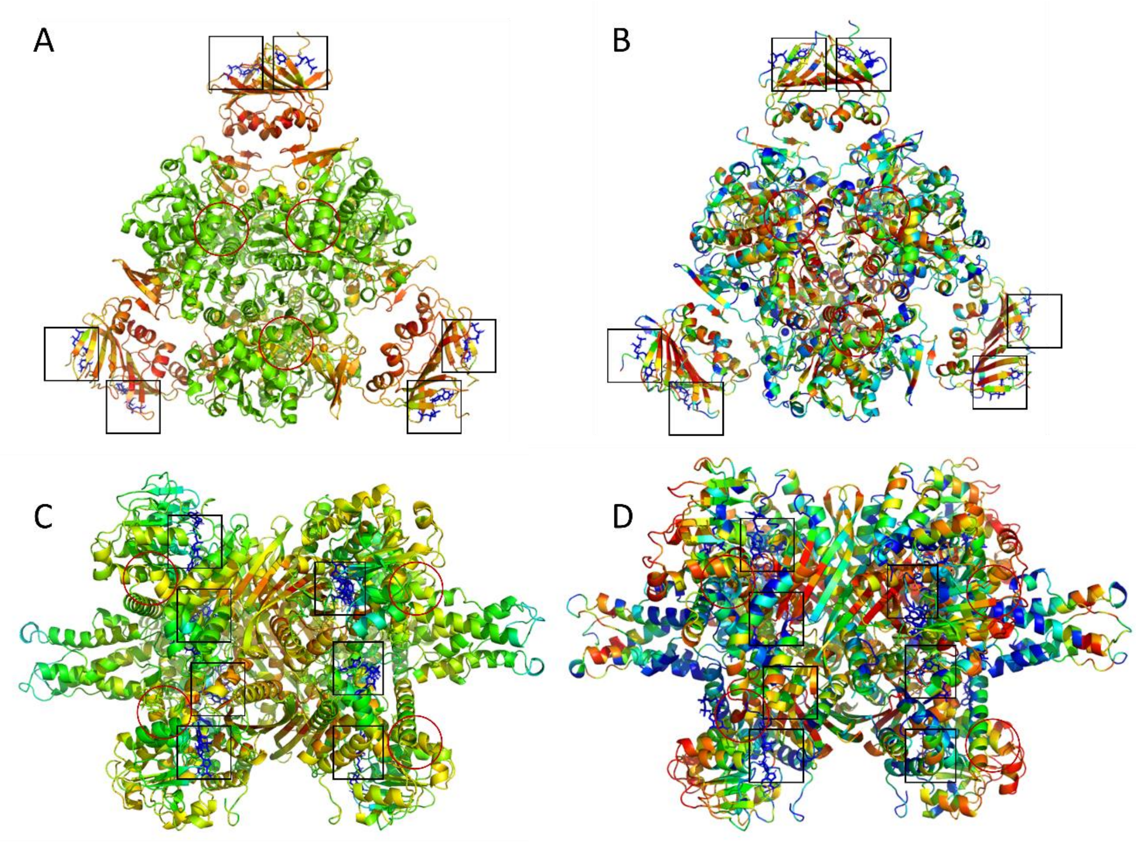

Supplementary Figure 23. Comparison of ACI of residues in 1D09 and 6DHD predicted by Ohm and Amor's method, respectively. (A) ACI in 1D09 predicted by Ohm. (B) ACI in 1D09 predicted by Amor's method. (C) ACI in 6DHD predicted by Ohm. (D) ACI in 6DHD predicted by Amor's method. Active sites are in black boxes and allosteric sites are in red circles.

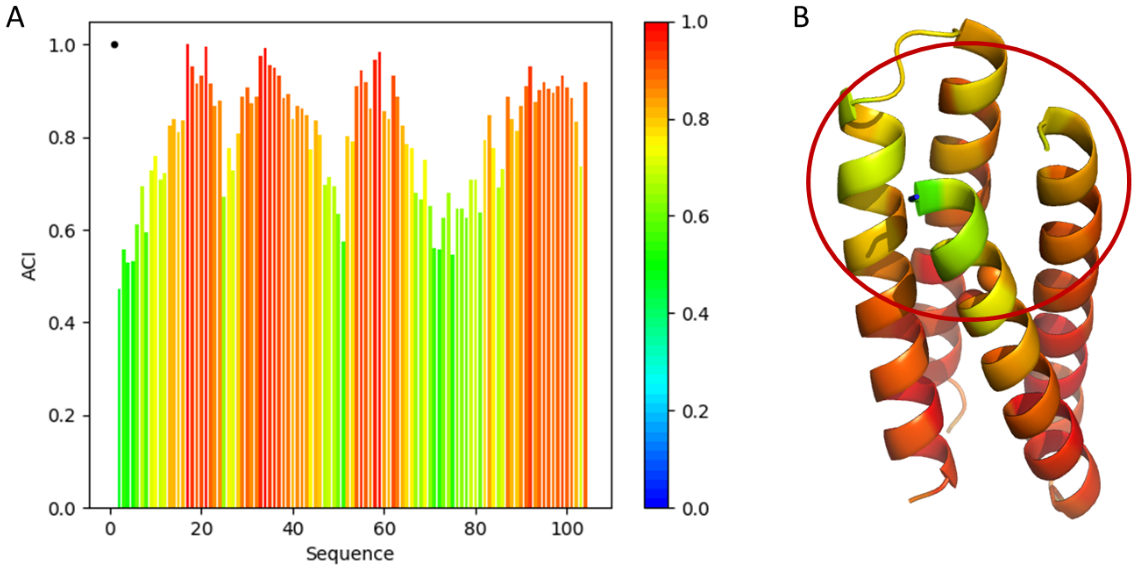

Supplementary Figure 24. ACI of residues in 1MFT. (A) The ACI of each residue in the protein. The black dot refers to the start point of the perturbation propagation algorithm. (B) The tertiary structure rendered by ACI. The red circle refers to the start point of the perturbation propagation algorithm.

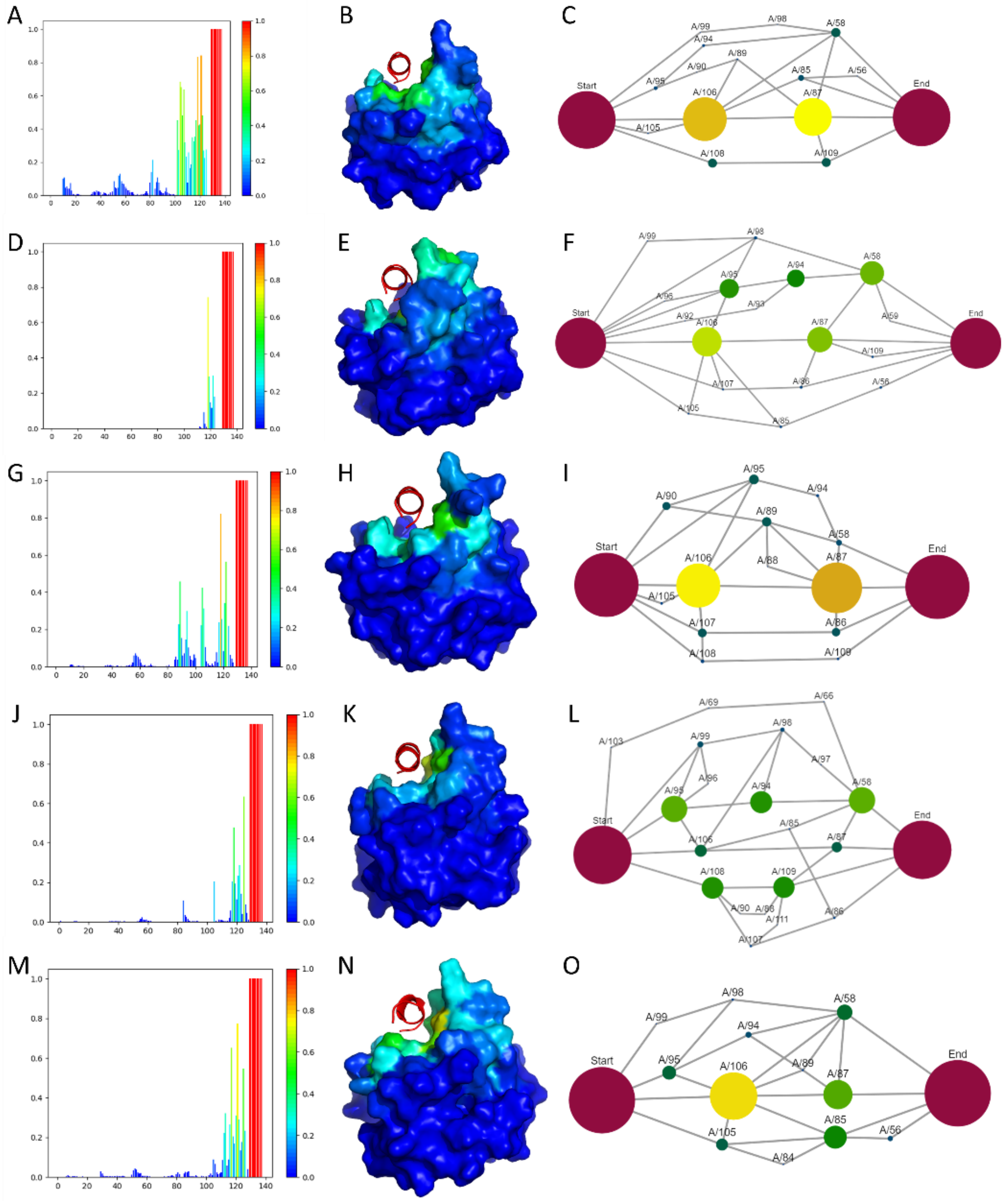

Supplementary Figure 25. Allosteric analysis of CheY at different MD simulation time. We use Gromacs to perform 100 ns simulation for CheY. At 0 ns (A, B, C), 25 ns (D, E, F), 50 ns (G, H, I), 75 ns (J, K, L), and 100 ns (M, N, O), the allosteric network of CheY changes moderately. The RMSD is 0 Å, 2.52 Å, 2.73 Å, 2.31 Å, and 2.30 Å with respective to the native structure, respectively.

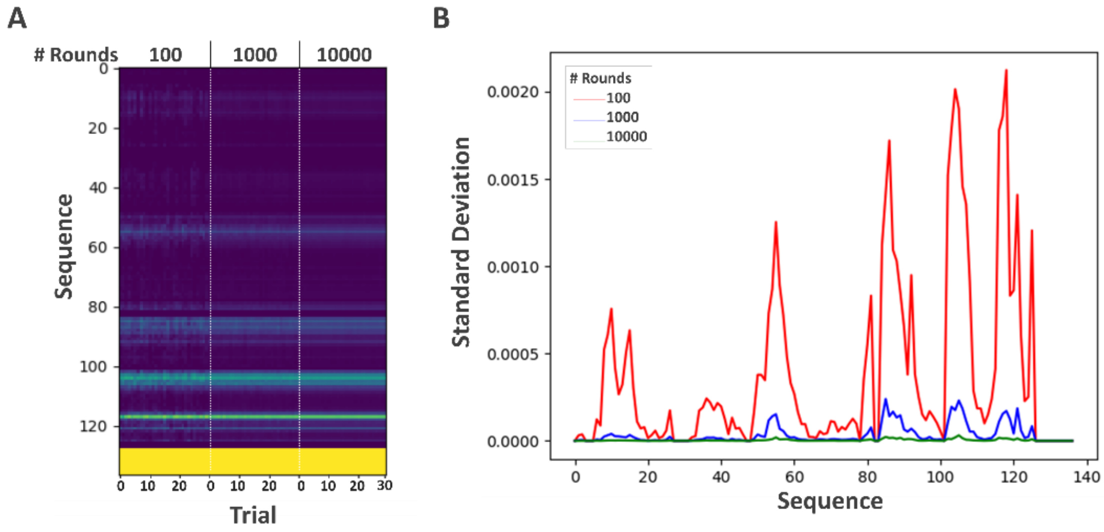

Supplementary Figure 26. Influence of the number of perturbation propagation rounds on the calculation of ACI. (A) The ACI of residues in CheY calculated by using different number of perturbation propagation rounds: 100, 1000, and 10000. We perform 30 trials for each number of rounds. (B) The standard deviation of ACI at each residue position.

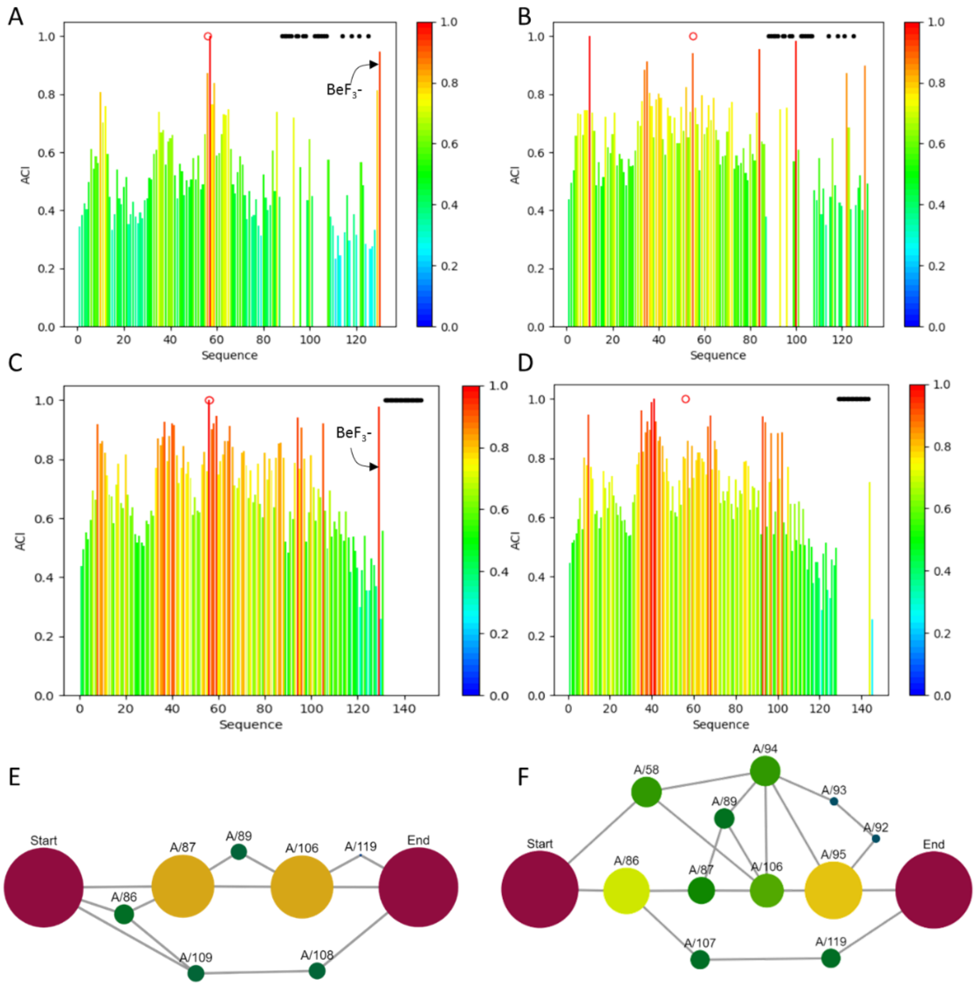

Supplementary Figure 27. Comparison of ACI by using different experimental structures of CheY. (A) ACI in 1FQW (*apo* CheY-BeF_3^-). (B) ACI in 3CHY (*apo* unphosphorylated CheY). (C) ACI in 1F4V (CheY-BeF_3-FliM). (D) ACI in 2B1J (unphosphorylated CheY-FliM). (E) Critical residues in 1F4V. (F) Critical residues in 2B1J. Black dots refer to the active sites and red circles refer to the allosteric sites.

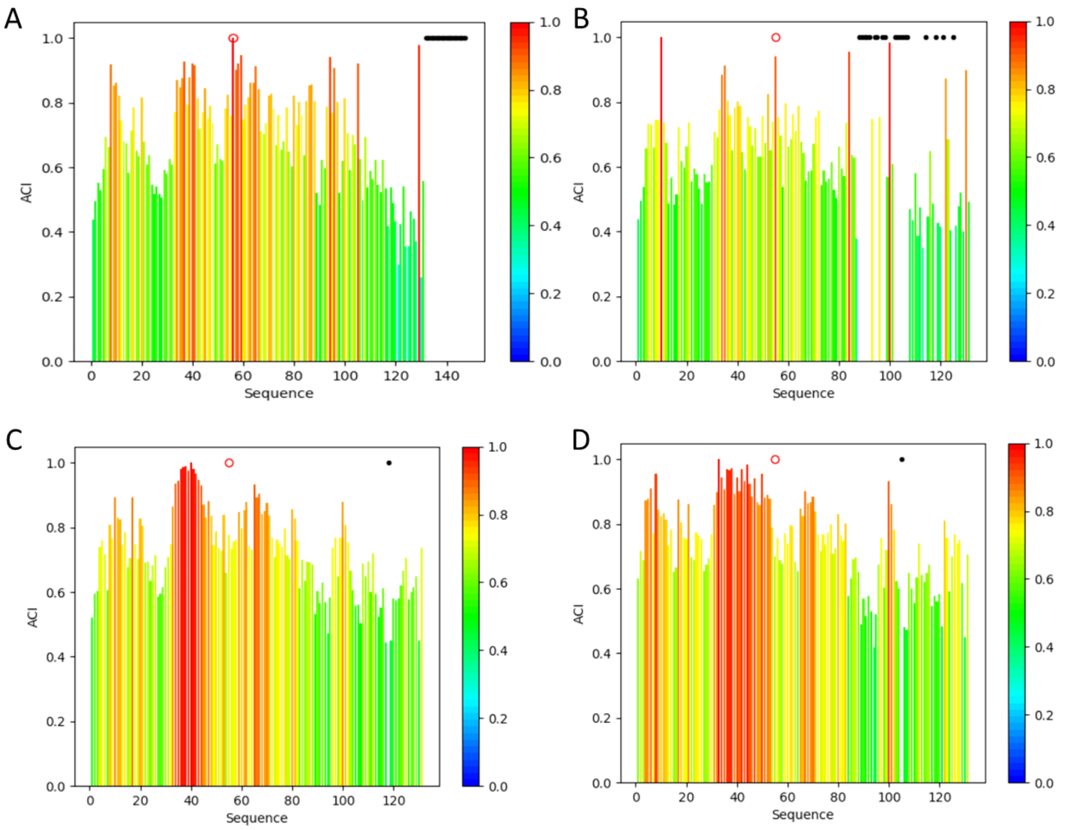

Supplementary Figure 28. Comparison of ACI in bound and unbound CheY when different residues are using as the start of the propagation algorithm. By using FliM as the start for the bound structure (A), or using all binding surface residues as the start for the unbound CheY structure (B), we can successfully find the allosteric site D57. However, if we choose Y106 (C) or K119 (D) for unbound CheY structure, we cannot find the allosteric site D57. Black dots indicate the position of active site residues and red circles refer to the active site D57.

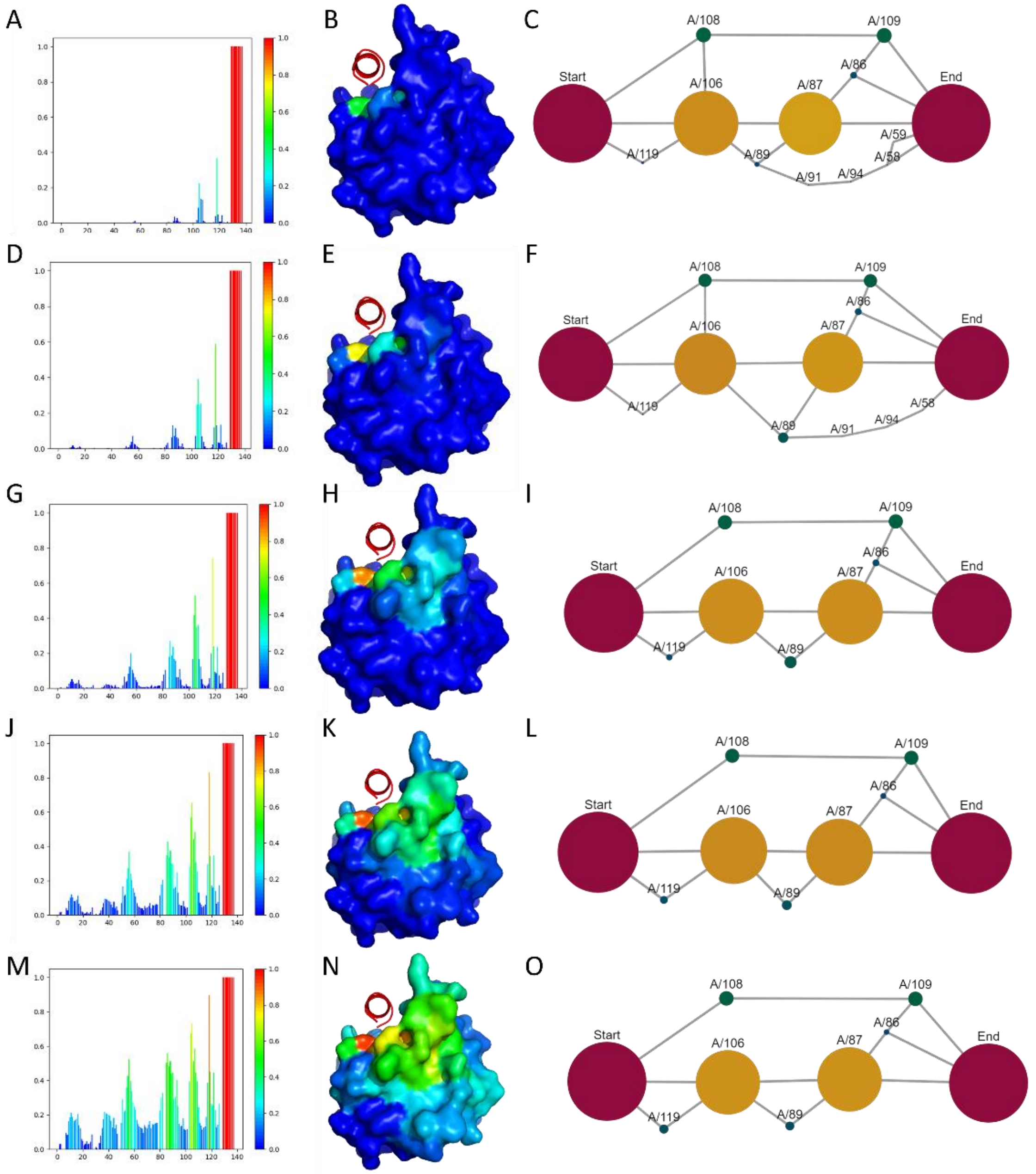

Supplementary Figure 29. Influence of α on allosteric network. We use different values of α to perform allosteric analysis for CheY: 1 (A, B, C), 2 (D, E, F), 3 (G, H, I), 4 (J, K, L), and 5 (M, N, O). A, D, G, J, and M are the ACI values calculated by using different α, respectively. B, E, H, K, and N are the tertiary structures rendered by ACI calculated with different α, respectively. C, F, I, L, and O are critical allosteric residues predicted with different α, respectively.

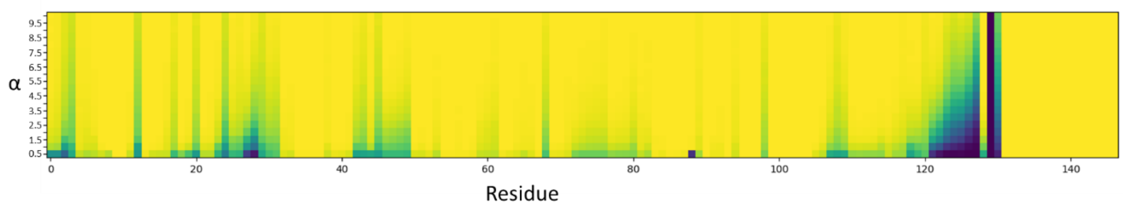

Supplementary Figure 30. ACI of residues in CheY calculated by different α. The x-axis refers to the residues and the y-axis is the value of α.

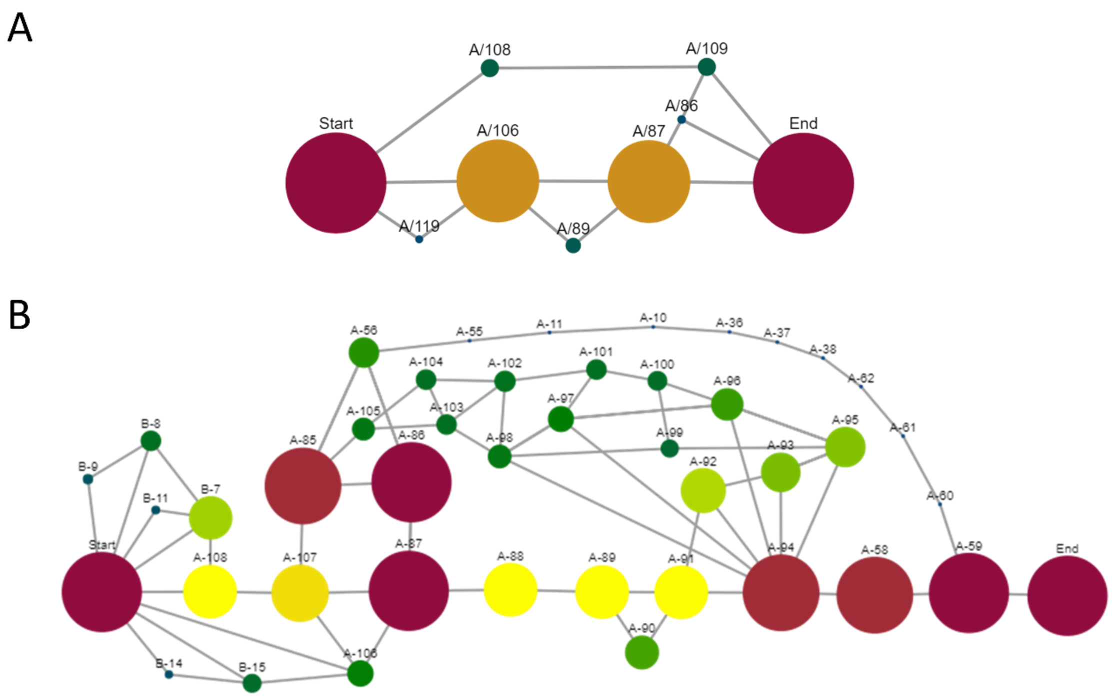

Supplementary Figure 31. Allosteric networks composed of critical residues of CheY predicted by Ohm with and without backbone-backbone contacts. (A) The allosteric network predicted by Ohm in the absence of backbone-backbone contacts. (B) The allosteric network predicted by Ohm in the presence of backbone-backbone contacts.

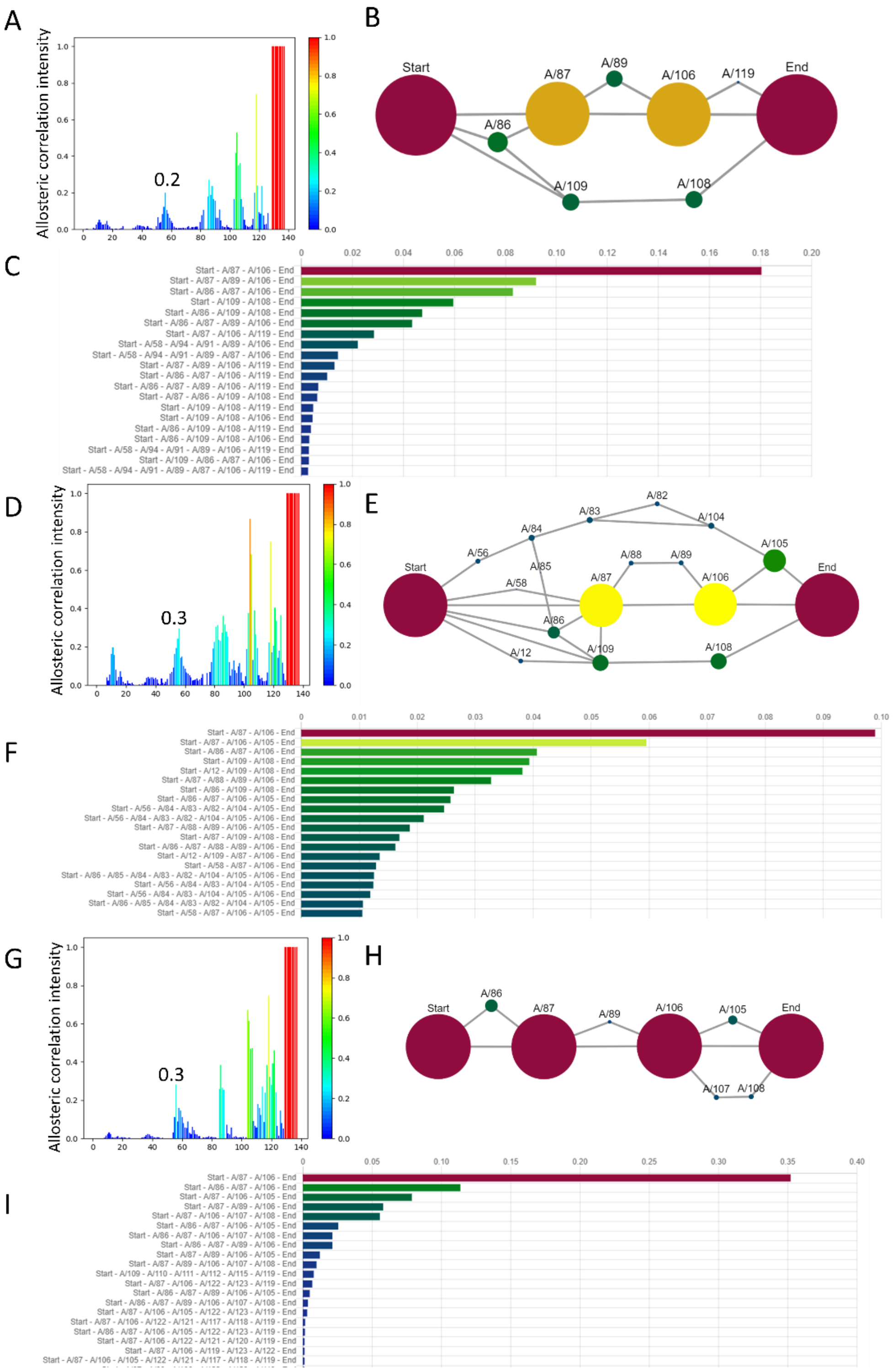

Supplementary Figure 32. Allosteric analysis of CheY wild type and mutants. We calculate ACI, identify allosteric pathways, and determine critical allosteric residues of wild type (A, B, C),

I55V (D, E, F), and V21A of CheY (G, H, I). The RMSD of I55V to the wild type is 1.63 Å, and the RMSD of V21A to the wild type is 1.50 Å.

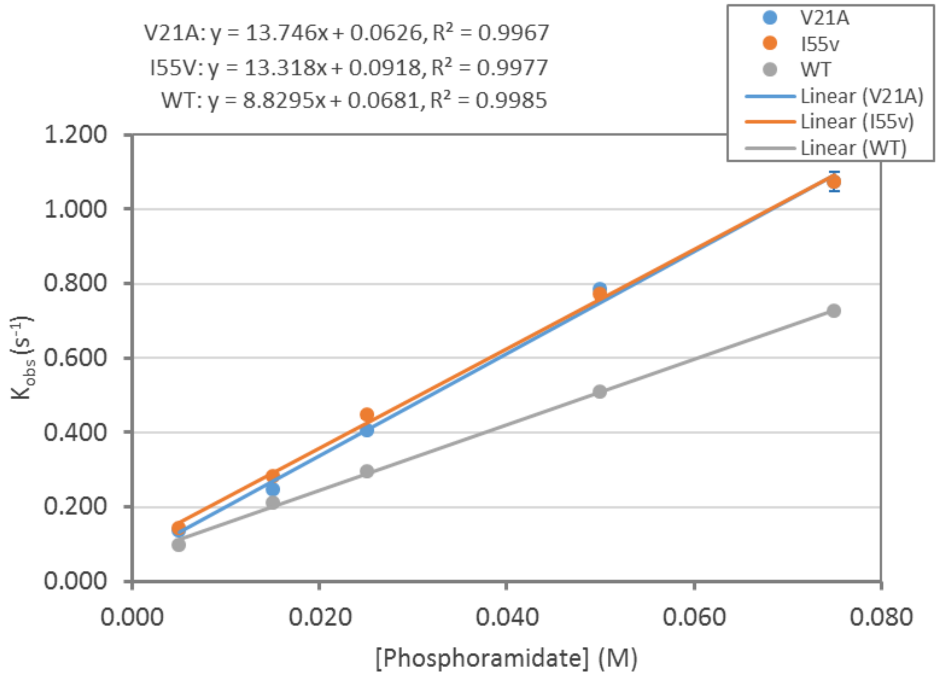

Supplementary Figure 33. The Autophosphorylation assay of CheY.

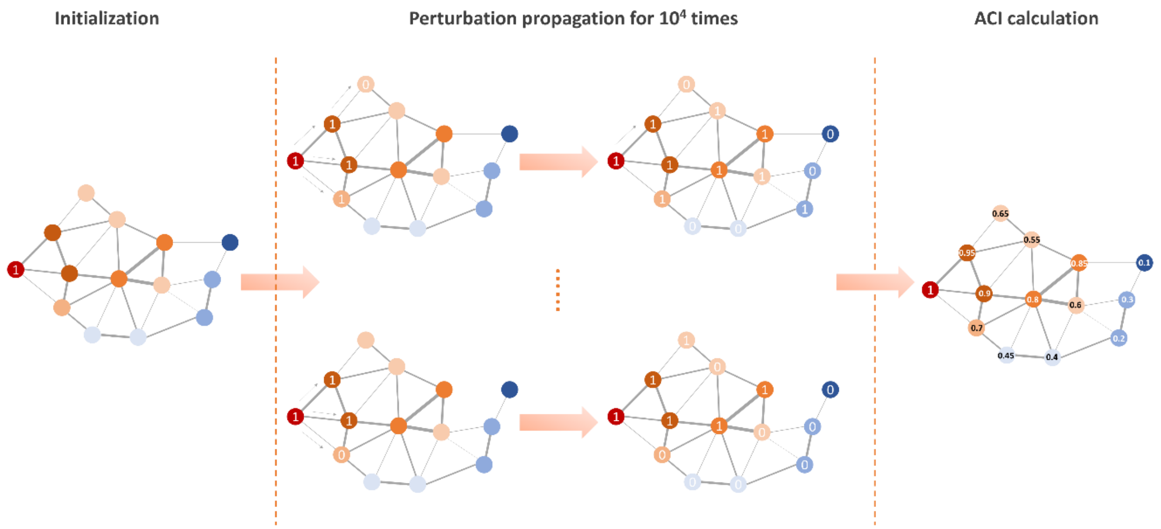

Supplementary Figure 34. The schematic diagram of the perturbation propagation process. Initially, each of the residues in the active site is assigned 1. Next, each neighbor residue of the active site residues is assigned 1 or 0 by the probability of the edge between them. Iterate this step until each residue is assigned a value. Finally, repeat the whole process 10000 times to calculate the frequency of each residue being assigned 1. Red colored nodes have final ACI close to 1 and blue colored nodes have final ACI close to 0.
