# Supplementary material for: Mapping allosteric communications within individual proteins
Source: Nat Commun. 2020 Jul 31;11:3862. doi: 10.1038/s41467-020-17618-2 (PMC7395124; doi:10.1038/s41467-020-17618-2)
Supplement: Supplementary file 3 — Description of Additional Supplementary Files [file 41467_2020_17618_MOESM3_ESM.docx]

**Description of Additional Supplementary Files**

**File Name**: Supplementary Data 1

**Description**: The fluorescence data of CheY. We provide the fluorescence intensities of residues in V21A, I55V, and the wild type CheY.

**File Name**: Supplementary Data 2

**Description**: The NMR data of the 7 mutations of CheY for CHESCA analysis. We provide the chemical shifts of unphosphorylated wild-type CheY and the seven mutants, F8V, D13K, M17A, V21H, T87I, Y106W, and A113P.

**File Name**: Supplementary Data 3

**Description**: Analyses results of Ohm in the 20 proteins data set. For each protein, we provide the ACI values of all residues, the active site residues, the allosteric site residues, the hotspots identified by ACI values, the predicted allosteric pathways, the allosteric network, the contacts, and the predicted residue correlations.

**File Name**: Supplementary Data 4

**Description**: Comparison of Ohm to Amor’s method. For each protein, we provide the quantile scores of Amor’s method and the hotspots identified by the quantile scores through the clustering method introduced in our manuscript.

**File Name**: Supplementary Data 5

**Description**: Comparison of different computational methods to NMR CHESCA analysis of residue correlation in CheY. The correlation data calculated by Ohm, PRS, minimum-distances (shortest paths), contacts, and hitting time are all provided and can be compared through the “compare.sh” script.
